# Effect of a 3-week program of cane training and use on gait of individuals with Parkinson’s disease: Protocol for a randomized controlled trial

**DOI:** 10.1371/journal.pone.0341248

**Published:** 2026-04-16

**Authors:** Jordana de Paula Magalhães, Merrill R. Landers, Aline Alvim Scianni, Maria Eduarda Bueno Santos Ribeiro, Christina Danielli Coelho de Morais Faria

**Affiliations:** 1 Department of Physical Therapy, Universidade Federal de Minas Gerais, Belo Horizonte, Minas Gerais, Brazil; 2 Department of Physical Therapy, University of Nevada, Las Vegas, Nevada, United States of America; Aichi Prefectural Mikawa Aoitori Medical and Rehabilitation Center for Developmental Disabilities, JAPAN

## Abstract

**Introduction:**

Although studies examining the immediate effects of cane use in individuals with Parkinson’s disease (PD) have reported negative outcomes, the efficacy and safety of structured training programs for assistive device use remain to be investigated. Furthermore, the limited evidence regarding patient-reported outcomes hinders the implementation of patient-centered, evidence-based practices in prescribing assistive devices for this population.

**Objective:**

To investigate the effect of cane training and use on gait speed (primary outcome), gait confidence, cadence, step length, functional mobility, freezing of gait, fear of falls, and satisfaction with the use of a cane (secondary outcomes) in individuals with PD.

**Materials and methods:**

A double-blind, randomized controlled trial will be carried out. A total of 26 individuals with PD will be randomly divided into two groups: (1) cane training and use (experimental group) or (2) global stretches and health education (time and attention controlled group). The intervention will be provided in four sessions lasting 40 minutes each, spaced over 15 to 22 days. Individuals will be instructed to use a cane (experimental group) or perform stretching exercises (time and attention-controlled group) daily, starting from the first day of training. Assessments will be conducted at the beginning of the study (week 0), post-intervention (week 3, post-intervention), and one month after the cessation of the intervention (week 8, follow-up). The primary outcome is gait speed. Secondary outcomes include gait confidence, cadence and step length, freezing of gait, functional mobility, fear of falls, and satisfaction with the use of cane. Between-group differences will be measured using a two-way repeated measures ANOVA, following intention-to-treat and per-protocol approaches (α=0.05),

**Discussion:**

The results of this study may improve the prescription of canes for individuals with PD. If effective, the cane could serve as a simple, low-cost, evidence-based intervention, and the training protocol could be replicated in clinical practice.

**Clinical trial registration:**

Effect of Training and Use of Cane on Gait in Individuals With Parkinson’s Disease - NCT06950255.

## Introduction

Parkinson’s disease (PD) is a neurodegenerative and progressive clinical condition. [1,2] Among the main neurological disorders, PD is the fastest growing in prevalence, disability, and deaths worldwide. [3] According to the Global Burden of Disease Study, it is estimated that by 2040 the number of individuals with PD will be approximately 13 million people globally, double the number of cases recorded in 2015. [4] This increase is mainly associated with the growth in the aging rate of the population. [4] Despite pharmacological treatment, the natural course of PD results in the progressive decline of various functions, compromising individuals’ participation in social, community, and family activities. [5–7]

The cardinal signs of PD (i.e., tremor, rigidity, bradykinesia, and postural instability) significantly affect the gait of individuals. [8] As a result, individuals with PD commonly present decreased gait speed, stride length, and swing time, along with increased cadence and double support time when compared with healthy individuals.(9) These gait impairments compromise the ability to move safely in different environments and often become noticeable with reduced gait speed, typically the first sign of gait alterations observed in this population. [5,7] Other gait alterations in individuals with PD typically include impaired postural balance and asymmetry between the limbs during gait. [8,9] Additionally, individuals with PD may experience hesitation to initiate gait and episodes of freezing, characterized by a sudden and brief stop of voluntary motor activity. [8,9] As the disease progresses, altered gait patterns also progress, increasing the risk of falls and affecting the mobility, independence, and quality of life of these individuals. [8,9]

A strategy commonly employed to improve the mobility of individuals with PD during gait involves the prescription of assistive devices, such as canes and walkers. [10] The use of these devices increases an individual’s base of support, allowing for a greater amplitude of movement for the center of mass. [10] Therefore, it is believed that the use of assistive devices can improve balance and confidence, and can also reduce falls during gait. [10] Furthermore, the use of these devices, as well as the perceived need for their use, tends to increase among individuals with PD over time. [11] However, the prescription for the use of these devices is based on the clinical judgment of healthcare professionals. No scientific guidelines or clinical guideline recommendations for the use of assistive devices for individuals with PD have been found. [10]

Few studies have been dedicated to investigating the effects of using assistive devices on the gait of individuals with PD. [12] In the study by Kegelmeyer et al. 27 individuals with PD were evaluated while walking with different assistive devices (single-point cane, standard walker, and two, four- and six-wheeled walker). [12] According to the authors, the use of these devices was associated with a decrease in step length, and swing phase, along with an increase in base of support and double support time. [12] Bryant et al. evaluated 10 individuals with PD while walking without an assistive device, with a single-point cane and with a walker. [13] The authors reported that the use of the cane reduced gait speed and the use of the walker decreased step length. [12] In the study conducted by Cubo et al. 19 individuals with PD were evaluated in walking activities without the use of assistive devices, with a standard walker and with a wheeled walker. [14] The authors identified that gait speed was greater without the use of the devices and the use of standard walker significantly increased the number and duration of freezing episodes. [14]

Although previous studies suggest that the use of assistive devices negatively impacts gait in individuals with PD, all studies investigated the immediate effects after the individuals received guidance and were familiarized with the devices just minutes before the final assessment. [12–14] Considering that individuals with PD often have difficulties with dual-task performance [15] and that motor learning requires repetitive, practice-based training [16] the efficacy and safety of structured training programs for the use of assistive devices need to be investigated. In a pilot study conducted by Martini et al., a training program had positive effects in preventing falls and reducing sitting time in individuals with multiple sclerosis who were already utilizing assistive devices. [17] The study protocol involved device adjustment, adaptation, and task-oriented training of walking with the assistive device in different circumstances. [17] In this study, training was offered in six sessions and the participants were followed up for up to three months post-intervention. [17]

Moreover, the previous studies on PD have predominantly concentrated on evaluating performance-based outcomes, [12–14] neglecting patient-centered outcomes related to perception, satisfaction, and adherence to the use of assistive devices. [18] Thus, the primary objective of this study is to investigate the effect of cane training and use on gait speed in individuals with PD. The secondary objective is to evaluate the effects of cane training and use on gait confidence, cadence, step length, functional mobility, freezing of gait, fear of falls, and satisfaction of individuals with PD regarding the use of a cane.

## Materials and methods

**Fig 1:**
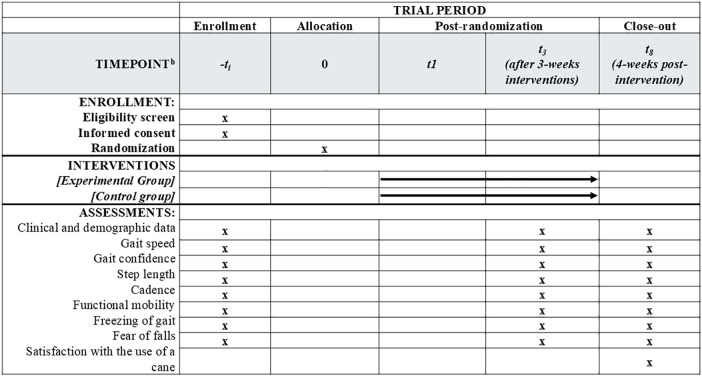
This is the Fig 1 Title: Participant timeline: Schedule of enrollment, interventions, and assessments.

**Fig 2:**
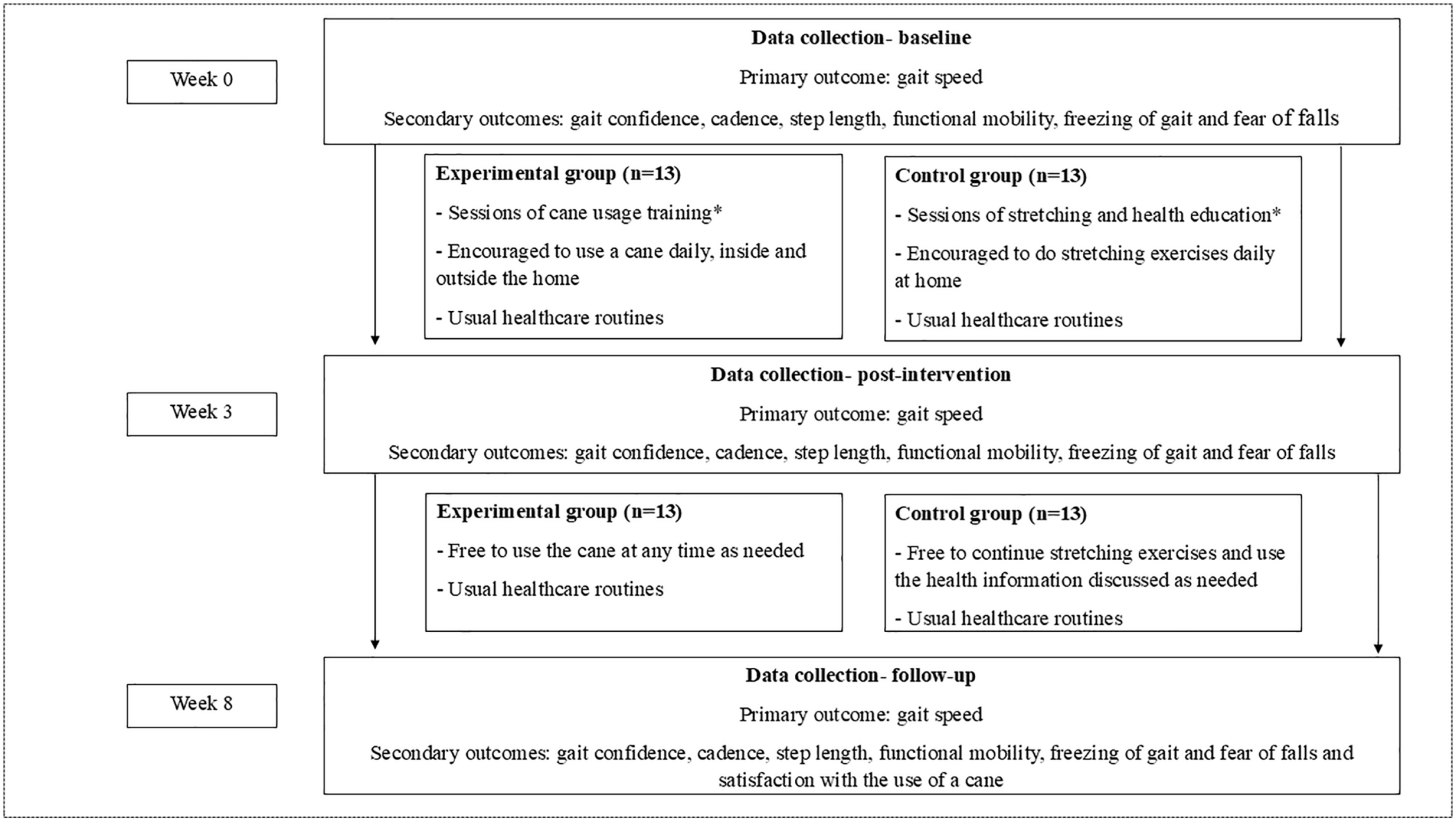
This is the Fig 2 Title: Design of the trial.

A prospective, exploratory parallel-group, double-blind randomized controlled trial, will be conducted and will be reported in accordance with the Consolidated Standards of Reporting Trials (CONSORT) guidelines. [19] Both treatment allocation and assessments will be blinded. Intention-to-treat and per-protocol analysis will be performed. Individuals with PD will be recruited in a community setting and randomly assigned to either the (1) cane training and use (experimental group) or (2) global stretches and health education (time and attention-controlled group). Assessments will be conducted at the beginning of the study (week 0), post-intervention (week 3, post-intervention), and one month after the cessation of the intervention (week 8, follow-up) (Fig 1). [20] The examiner responsible for assessments will be blinded to group allocation, and participants and the treating physiotherapist will be asked not to share any information about the intervention with the examiner (Fig 2). Group assessments and training will be carried out in different places and on different days. This randomized controlled trial was prospectively registered at ClinicalTrials.gov (NCT06950255) and received ethical approval from Institutional Research Ethics Committee (CAAE: 75158123.2.0000.5149). Participant recruitment began in May 1^st^, 2025 and is expected to be completed in September 1^st^, 2026.

This is the Fig 2 legend. *Four sessions over 15–22 days

### Participants

A non-probabilistic sample will be recruited from the community in the city of Belo Horizonte, Minas Gerais, Brazil. Recruitment will involve lists of participants from other research/extension projects and dissemination through social media, health centers, and the university. All subjects will be informed about the study procedures and will provide written consent. Individuals will be included according to the following criteria: age ≥ 40 years; diagnosis of idiopathic PD confirmed by a neurologist; classification between stages II to IV of the modified Hoehn & Yahr Scale (HY); [21] use of anti-parkinsonian medication with stable pharmacological therapy for at least 6 months; ability to walk independently in a 14-meter corridor with a gait speed ≤ 1.1 m/s (defined to screen individuals with mobility impairment); and, ability to use a single-point cane during walking correctly and safely with no regular use of any type of assistive device since the diagnosis of PD.

Individuals with cognitive impairment, assessed by the Mini Mental State Examination, [22] those using deep brain stimulation, or those who present any other neurological, cardiopulmonary or musculoskeletal condition that may compromise the test performance will be excluded.

### Randomization

After baseline assessments, individuals will be randomly allocated into the experimental and control groups. The allocation sequence will be concealed within sealed opaque envelopes prepared before the commencement of the study by a research assistant not involved in the study.

### Patient and public involvement in this study

Two individuals with PD were involved as collaborators in this study to contribute to the relevance of the research through the inclusion of patient perspectives. [23] The two collaborators were chosen because of their interest and availability. During the planning of the study, these collaborators contributed to clarifying the research question and its importance to patients, and helped to review the research methods, data collection, and recruitment. [23] It is expected that these collaborators will be involved in future stages of interpretation of the results, adding their opinions based on their lived experiences with PD. [23] Additionally, these individuals will be involved in the production of scientific products to disseminate the research results. [23]

### Procedures

All participants will be informed of the study procedures and will provide written consent under Institutional Review Board approval. A trained researcher, blinded to the group allocation, will collect all measures and sociodemographic and clinical/functional data to characterize the sample. After the data collection, participants will be randomly allocated to either the experimental or control group by the physiotherapist responsible for the intervention. This physiotherapist will be trained and blinded to the evaluation results of the participants. After allocation, all individuals will be instructed to maintain their usual healthcare routines. To ensure the integrity of blinding procedures, participants will be explicitly instructed not to discuss any aspect of the intervention during post-intervention assessments. Furthermore, all participants will attend the assessment sessions without a cane. During the assessments, a standard cane, identical to the one provided to the experimental group, will be used exclusively for testing. All participants will perform the performance tests with and without the cane, in a random order. Data on the success of blinding will be collected and reported.

### Experimental group

After allocation, individuals in the experimental group will receive cane usage training, provided in four sessions lasting 40 minutes each, spaced over a period of 15–22 days. The intervention protocol was based on the recommendations that training in the use of assistive devices should involve multiple sessions and that patients should be reassessed after approximately two weeks of using the device. [24] A flexible session schedule was adopted to optimize participant recruitment and retention, as recommended. [25] In addition to the training sessions, individuals will be instructed and encouraged to use the cane in their daily mobility activities, both indoors and outdoors, starting from the first day of training. To monitor adherence, individuals will receive a diary to record on which occasions and for how long they used the cane during mobility activities. The diaries were structured with daily checkboxes in a Likert-type format to record cane use as: no time (0%), a small portion of the time (around 25%), some of the time (around 50%), most of the time (around 75%), or all the time (around 100%). To enhance adherence, participants will receive telephone follow-ups between sessions aimed at monitoring and encouraging device use.

Individuals will receive a single-point cane, made of aluminum, with an ergonomic handle and 10 adjustment levels. The height of the cane will be individually adjusted to maintain each individual’s elbow at approximately 30 degrees of flexion. [13] Participants will be instructed to use the device on their dominant side or the side with less impairment. [26] The training with the cane will be carried out by a trained physiotherapist. [27] The training protocol will include gait training with the cane on different surfaces and at different speeds, divided into the following stages:

1. Ground walking at a comfortable speed on a flat, stable surface – 5 minutes.
2. Ground walking at a comfortable speed on a steep, stable surface (ramp) – 10 minutes.
3. Ground walking at maximum speed on a flat, stable surface – 10 minutes.
4. Ground walking at a comfortable speed on a flat, stable surface – 5 minutes.

An interval will be provided for the rest between stages and as necessary. In addition, at the beginning of each training session, individuals will have the opportunity to address any questions they may have about using a cane in their daily life context such as on uneven ground and stairs (10 minutes).

Individuals will be re-evaluated after the end of the training sessions. After re-evaluation, the canes will be left with participants who will be instructed as follows: “Feel free to use the cane at any time as needed.” Another diary will be provided for the participant to record the use of the device after the end of the intervention.

### Time and attention control group

To ensure a comparable amount of attention received, individuals in the control group will receive an intervention involving global stretching of the upper and lower limbs and health education. [28,29] The intervention will be provided in four sessions lasting 40 minutes each, spaced over 15–22 days. Additionally, individuals allocated to the control group will be instructed not to start using any assistive device during the study period and encouraged to perform stretching daily at home from the first day of training. To monitor adherence, individuals will receive a diary to record stretching exercises performed daily. To enhance adherence, participants will receive telephone follow-ups between sessions aimed at monitoring and encouraging the performance of the stretching exercises.

The intervention for the control group will consist of 20 minutes of global static stretching and 20 minutes of guidance on general health care. The stretches will be performed in three sets of 30 seconds each, targeting different muscle groups. [28,29] If a participant is unable to perform the self-stretches, the researchers responsible for implementing the intervention will provide assistance. The health education component will cover information on PD and fall prevention, following the Portuguese version of the European Physiotherapy Guideline for PD. [30]

The intervention will be offered by the same physiotherapist responsible for training the experimental group. Individuals will be re-evaluated after the end of the training sessions. After re-evaluation, the participants will be instructed as follows: “Feel free to continue stretching exercises and use the health information discussed as needed.” Another diary will be provided for the participant to record the stretching exercises performed after the end of the intervention.

### Outcome measures

#### Primary outcome measure.

Gait speed

Gait speed will be measured by the 10-Meter Walk Test (10MWT). The 10MWT is a performance-based test used to assess gait speed in a 14-meter corridor. [31] The central 10 meters will be used to calculate walking speed, with the initial two meters and the final two meters disregarded to account for acceleration and deceleration. [31] Participants will be instructed to walk at their usual speed. This test has adequate measurement properties for evaluating this outcome in individuals with PD and is widely used in clinical practice. [31] A preliminary test will be conducted for participant familiarization, followed by a subsequent test to record the measurement. [32] In the case of freezing episodes during the test, the number of episodes and freezing duration will be recorded. [2]

#### Secondary outcome measures.

Gait confidence

Gait confidence will be assessed using the Gait Efficacy Scale-Brazil (mGES-Brazil). [33] The mGES-Brazil is a ten-item scale that assesses an individual’s confidence while walking in challenging circumstances, such as walking on different surfaces. [33] Each item on the scale is individually scored on a Likert scale ranging from zero to ten points, with higher scores indicating greater confidence. [33] The scale has already been cross-culturally adapted to Brazilian-Portuguese and demonstrated adequate measurement properties for evaluating gait confidence. [33]

Step length and cadence

Step length and cadence will be assessed during the 10MWT. To measure step length and cadence, the number of steps taken during the 10MWT will be counted following previous procedures. [34] To calculate step length, the distance walked, i.e., 10 meters, will be divided by the number of steps taken. [30,34,35] Cadence will be determined by dividing the number of steps by the time taken to complete the 10MWT. [30,34,35] The use of 10MWT to assess step length and cadence has been widely used in the literature and recommended by clinical guidelines for this population. [30,35]

Functional mobility

Functional mobility will be assessed using the Timed “Up and Go” test (TUG). The TUG is an easy-to-apply test that measures the time taken by the individual to get up from a chair, walk 3 meters, turn around and return to the chair. [30] This test has adequate measurement properties for evaluating functional mobility in individuals with PD. [30] A preliminary test will be conducted for participant familiarization. Subsequently, the test will be performed again, and the time of the second test will be recorded. [30] As with the gait speed test, the number and duration of possible freezing episodes will be recorded. [2]

Freezing of Gait

Freezing of gait will be assessed by the Freezing of Gait Questionnaire (FOG-Q). This questionnaire has six items aimed at evaluating freezing during gait in participants with PD. [36,37] The instrument has already been translated and cross-culturally adapted into Brazilian Portuguese. [36] Furthermore, the questionnaire has adequate measurement properties and is considered reliable for tracking and measuring the severity of freezing, as well as for evaluating this outcome after interventions. [36] In addition to the questionnaire, the number and duration of freezing episodes that participants experience during the 10MWT and the TUG will be reported. The tests will be video recorded to allow detailed analysis of freezing episodes during the assessment. The records will be evaluated by the same assessor responsible for the other outcome measures, who will remain blinded to group allocation.

Fear of falls

Fear of falls will be assessed by the Falls Efficacy Scale – International (FES-I). This questionnaire has 16 items that assess the individual’s concerns about the possibility of falling while carrying out different daily tasks. [38,39]Each item of the questionnaire is scored from one to four, where higher scores reflect greater concern. [38,39] This scale has already been cross-culturally adapted to Brazilian-Portuguese and presents adequate validity and reliability for assessing this outcome in individuals with PD. [38,39]

Satisfaction with using the cane

Satisfaction regarding the use of the cane will be assessed by the Quebec User Evaluation of Satisfaction with Assistive Technology (QUEST 2.0). [40] The questionnaire has 12 items, scored from one to five, where higher scores reflect greater participant satisfaction with assistive technology. [40] This instrument has already been translated and cross-culturally adapted into Brazilian Portuguese and has proven to be reliable and valid for measuring the satisfaction of users of assistive gait technology. [40] Satisfaction with cane use will be assessed only in participants from the experimental group at the end of the study.

### Adverse events

Adverse events will be monitored during the intervention period. In this study, an adverse event will be defined as any untoward medical occurrence associated with intervention – such as pain, falls, or exercise intolerance – that necessitated hospitalization or further treatment. [41,42]

### Sample estimation

Sample calculation was performed using the minimal clinically important difference (MCID) and the standard deviation of individuals with PD in the 10MWT. According to previous studies, a change of 0.22 m/s is considered as a MCID for gait speed and a minimal detectable change in the 10MWT in PD. [43,44] In this population, the standard deviation found in the 10MWT was 0.15 m/s. [44] Considering a significance level of 5% and a power of 0.80, 18 participants are required. Sample loss was calculated using the formula 1 ÷ (1 – d), where d is the dropout rate. [45] We adopted a conservative dropout rate of 30%, based on previous studies involving individuals with neurological conditions in the same region [46–48] and considering the well-documented challenges faced by individuals with PD, particularly those in more advanced stages in maintaining participation throughout research studies. [25] Subsequently, a final sample size of 26 individuals was determined (13 in each group).

### Data analysis

The analysis will be carried out by an independent researcher, blinded to the randomization of the groups, using intention-to-treat and per-protocol analysis. For the per-protocol analyses, we will include individuals who complete at least three training sessions (a 75% attendance rate) and who use the device for approximately 75% of the time during their daily routine activities throughout the training period. Data normality will be tested for all continuous numerical variables. Descriptive statistics will be used to characterize the sample and investigate satisfaction with the use of a cane. The difference between the groups for the other outcome variables (gait speed, gait confidence, cadence, step length, functional mobility, freezing of gait, and the number of freezing episodes during the 10MWT and TUG) will be assessed using mixed factorial 2 (group: experimental and control) X 3 (time: baseline, post, follow-up) ANOVA on all of the aforementioned outcome measures. Demographics and/or clinical functional variables that have been shown to relate to the dependent variable will be explored for potential inclusion as a covariate. All statistical analyses will be performed using SPSS statistical software (Version 23.0 SPSS Inc., Chicago, IL, United States). For all inferential analyzes, a significance level of α = 0.05 will be used.

## Discussion

Assistive devices are commonly recommended for individuals with PD in an attempt to maintain independence for as long as possible. [49] According to previous studies, the percentage of use of these devices can reach 66% of individuals with PD, with the cane being one of the assistive devices most used by this population. [10,11] Although canes are used by individuals with PD, the effects of using this assistive device on gait are poorly understood. [49] Previous experimental studies on this topic only evaluated the immediate effects of using these devices, and the training and use by participants occurred only minutes before data collection. [12–14] Additionally, study participants could only experience the use of the device in a clinical context. [12–14] Therefore, the results of previous studies are insufficient to provide evidence of the effect of the cane on the gait of individuals with PD, as motor learning requires repeated and context-specific practice of a motor task. [50] The results of the present study will be innovative as individuals will have the opportunity to train using the cane in a guided, supervised, and repetitive manner and will use the cane in their real-life context for a longer period of time.

Furthermore, the outcomes of previous studies focused primarily on gait parameters such as gait speed, cadence, and step length. [12,13]. Although these data are important in identifying altered gait patterns, they do not provide information on other important outcomes that may impact mobility during gait, such as confidence to walk in different environments and the occurrence and severity of freezing of gait. [5,12,13] Therefore, the results of the present study will add important information about the effects of the cane on mobility-related outcomes that have not yet been investigated. Finally, although canes are prescribed in clinical practice, satisfaction with the use of canes by individuals with PD is unknown. This information could improve understanding of individuals’ perceptions of using the device. Overall, the results of the present study could enhance the prescription of cane use for individuals with PD.

In this study, a single-point cane will be used. In addition to being a commonly used device by individuals with PD, [11] the single-point cane is lightweight, inexpensive, and durable, which contributes to the clinical utility of this device. [51] The use of a cane can increase the base of support and this helps with dynamic balance during gait. [24,51] In addition, the cane is the least restrictive of all assistive devices. [24,51] Thus, it is possible that training and use of the cane may improve mobility during gait. However, no previous clinical trials have investigated this hypothesis in individuals with PD.

Although research on the non-pharmacological treatment of motor symptoms of PD has advanced in recent years, the implementation of evidence-based practice still faces many challenges given the lack of scientific support for several interventions used to treat this population. [52] Regarding the investigation of the effects of using canes in individuals with PD, no controlled clinical trials were found. Therefore, although assistive devices have been used by individuals with PD and recommended by clinical guidelines, [11,30,35,53] there is no current scientific evidence to support their use or prescription. Research shows that evidence-based practice is associated with improvements in outcomes, reduced costs, and attribution of value to healthcare. [54] Therefore, the results of the present study will provide initial information that could improve the cost and effectiveness of care for this population.

In summary, findings of the present study may improve the prescription of canes for individuals with PD. If the results show the efficacy and safety of the investigated protocol, the cane could be used efficiently, that is, based on evidence, as a simple, low-cost and low-maintenance intervention. In clinical practice, the training protocol investigated in this study could be replicated by healthcare professionals based on the outcomes observed. Moreover, the findings may support patient education regarding adherence to cane use, based on the expectation of improvement and potential adverse effects of device use.

This study has limitations. Firstly, it will include a convenience sample, which may limit the generalizability of the results. Secondly, both the participants and the physiotherapist providing the interventions cannot be fully blinded as the intervention is readily observable. Furthermore, the present study will include individuals classified as stage II to IV on the Hoehn and Yahr scale. Since PD symptoms tend to worsen with disease progression, future studies should investigate the differential impact of disease severity on the efficacy and safety of cane use in individuals with PD. Finally, despite the availability of many cane and assistive device models, the study will provide data regarding a specific device. Future studies should investigate the effects of other devices on the gait of this population.

## Supporting information

S1 File Complete protocol approved by the ethics committee (in English). (PDF)

S2 File Complete protocol approved by the ethics committee (in Portuguese). (PDF)

S3 File SPIRIT checklist (DOCX)
