# Supplementary material for: Effect of a 3-week program of cane training and use on gait of individuals with Parkinson’s disease: Protocol for a randomized controlled trial
Source: PLoS One. 2026 Apr 16;21(4):e0341248. doi: 10.1371/journal.pone.0341248 (PMC13086423; doi:10.1371/journal.pone.0341248)
Supplement: S1 File — (PDF) [file pone.0341248.s001.pdf]

EFFECT OF TRAINING AND USE OF A CANE ON THE MOBILITY OF INDIVIDUALS WITH PARKINSON'S DISEASE: A RANDOMIZED CLINICAL TRIAL

Coordinating researcher: Christina Danielli Coelho de Morais Faria, Ph.D., associate professor in the Department of Physiotherapy at the Federal University of Minas Gerais (UFMG).

Collaborating researcher: Jordana de Paula Magalhães, M.Sc., doctoral student in the Postgraduate Program in Rehabilitation Sciences, UFMG.

Area of knowledge: Health Sciences

School of Physical Education, Physiotherapy and Occupational Therapy

Federal University of Minas Gerais

2023

RESEARCH PROJECT TEAM

1. Christina Danielli Coelho de Morais Faria (Brazil). Physiotherapist, PhD, associate professor, full-time, in the Department of Physiotherapy at the School of Physical Education, Physiotherapy and Occupational Therapy (EEFFTO) of the Federal University of Minas Gerais (UFMG). Accredited to the Postgraduate Program in Rehabilitation Sciences (PPGCR) at UFMG.

2. Jordana de Paula Magalhães (Brazil). Physiotherapist, MSc, doctoral candidate in the PPGCR program at UFMG under the supervision of Professor Christina Faria (UFMG), whose doctoral thesis will be developed using the results of this project.

INTRODUCTION

Parkinson's disease (PD) is a progressive, neurodegenerative clinical condition with increasing prevalence worldwide.(AASETH et al., 2018; BLOEM et al., 2021)Among the major neurological disorders, PD is the one that is growing most rapidly in prevalence, disability, and mortality.(GBD, 2017)According to the Global Burden of Disease Study, it is estimated that by 2040 the number of individuals diagnosed with PD will be approximately 13 million worldwide.(GBD, 2017; RAY DORSEY et al., 2018)This number represents double the number of cases recorded in 2015.GBD, 2017; RAY DORSEY et al., 2018)This indicates a rapid increase in the incidence and duration of the disease. This increase has been mainly associated with the rising rate of population aging.(RAY DORSEY et al., 2018)Therefore, advances in clinical research related to PD disorders are essential.

Recent studies on the pathogenesis of PD indicate that the onset of this health condition is associated with the abnormal deposition of protein aggregates rich in α-synuclein, called Lewy bodies, in the central nervous system.(BORGHAMMER, 2018; CÓPPOLA, 2018; RAZA et al., 2019)According to the staging scheme proposed by Braak et al. (2003), the first staging sites for PD are commonly the olfactory bulb, dorsal motor nucleus of the vagus nerve, and the lower region of the brainstem.(BORGHAMMER, 2018; BRAAK et al., 2003)Subsequently, the monoaminergic pontine nuclei and dopaminergic neurons of the substantia nigra are affected.(BORGHAMMER, 2018; BRAAK et al., 2003)This staging scheme explains the clinical evolution of PD, characterized by the initial appearance of non-motor symptoms that include alterations such as sleep disorders, hypothyroidism, and constipation.(SCHAPIRA et al., 2017).

The main motor symptoms of PD are known as cardinal signs and refer to resting tremor, bradykinesia, rigidity, and postural instability.(AASETH et al., 2018; SCHAPIRA et al., 2017). Those symptoms are predominantly caused by the loss of dopaminergic neurons in the substantia nigra pars compacta of the central nervous system.(SCHAPIRA et al., 2017) Therefore, one of the most used pharmacological therapeutic strategies in the treatment of PD is dopamine replacement, through the oral administration of the drug levodopa. (POEWE et al., 2020; SCHAPIRA et al., 2017)According to the diagnostic criteria adopted by The United Kingdom PD Society, the diagnosis of PD should be made in three stages: 1) Diagnosis of Parkinsonian

syndrome: presence of bradykinesia associated with another symptom among the cardinal signs. 2) Exclusion of other diagnoses that may cause Parkinsonian syndrome, such as stroke or traumatic brain injury. 3) Presence of three or more supporting criteria such as unilateral onset, resting tremor, progression of symptoms, and improvement of symptoms with the use of Levodopa.(MARSILI et al., 2018)As the disease progresses, Levodopa doses are commonly increased, resulting in periods known as the "on period," during which motor symptoms are attenuated, and the "off period," during which motor symptoms reappear.(SCHAPIRA et al., 2017)However, even with pharmacological treatment measures, the natural course of the disease results in the progressive decline of various functions in patients with PD, including mobility.(BOUCĄ-MACHADO et al., 2018; TOMLINSON et al., 2014)In these individuals, the decline in mobility compromises the ability to move safely in different environments and commonly becomes noticeable with a reduction in walking speed, usually the first sign of gait changes observed in this population.(BOUCA-MACHADO et al., 2018).

Individuals with PD commonly present with reduced gait speed, decreased step length, limb asymmetry, decreased upper limb balance, increased base of support and cadence, and reduced postural balance during gait.(DI BIASE et al., 2020; MIRELMAN et al., 2019)Furthermore, individuals with PD may exhibit hesitancy to initiate walking and episodes of freezing, characterized by a sudden, transient cessation of voluntary motor activity.(DI BIASE et al., 2020; MIRELMAN et al., 2019)As the disease progresses, altered gait patterns may also worsen, affecting mobility, independence, and quality of life for these individuals.(DI BIASE et al., 2020; MIRELMAN et al., 2019).

A strategy commonly used in an attempt to improve the mobility of individuals with PD during gait requires the prescription of assistive devices, such as canes and walkers.(BRYANT et al., 2014)The use of these devices increases the base of support for individuals, allowing for a greater range of movement of the center of mass during gait.(BRYANT et al., 2014)Therefore, it is believed that the use of these devices can improve balance, confidence, and reduce falls during walking.(BRYANT et al., 2014; KADER et al., 2018)Furthermore, the use of these devices, as well as the perceived need for their use, tends to increase among individuals with PD over the years.(KADER et al., 2018)However, the prescription of these devices is based solely on the clinical judgment of healthcare professionals, and no scientific guidelines on recommendations for the use of assistive devices for individuals with PD were found.(BRYANT et al., 2014).

In a study conducted with 85 individuals with PD, Bryant and colleagues (2014) identified that 36.6% of individuals used assistive devices while walking, with the cane being the most common device.(BRYANT et al., 2014)Furthermore, it was identified that 56.8% of individuals who took more than 13 seconds to complete the five-meter Timed Up Go test regularly used assistive devices. Among individuals who regularly used such devices, 77.8% showed lower levels of confidence in their balance.(BRYANT et al., 2014)However, the authors did not compare the individuals' performance in walking activities with and without a cane.(BRYANT et al., 2014)Furthermore, the study did not consider the average time the device was used by the individuals who reported using it.(BRYANT et al., 2014).

Other studies have focused on investigating the effects of using assistive devices during gait in patients with PD.(KEGELMEYER et al., 2013)Kegelmeyer et al. (2013) investigated the influence of the use of different assistive devices on gait patterns in 27 individuals with PD.(KEGELMEYER et al., 2013) According to the authors, only the use of the four-wheeled walker reproduced gait patterns similar to gait without the device.(KEGELMEYER et al., 2013)For the other devices evaluated (single-point cane, standard walker, and two- and six-wheeled walkers), their use decreased walking speed for all individuals.(KEGELMEYER et al., 2013)Furthermore, the use of these devices was associated with decreased step length, decreased base of support, increased double support time, and decreased swing phase during gait.(KEGELMEYER et al., 2013).

In another study, conducted by Bryant et al. (2012), 10 individuals with PD were evaluated during gait without an assistive device, with a single-point cane, and with a walker.(BRYANT et al., 2012)The authors reported that the use of the devices decreased walking speed.(BRYANT et al., 2012)Furthermore, the use of a walker decreased step length during gait. In a study conducted by Cubo et al. (2003), 19 individuals with PD were evaluated in gait activities without the use of assistive devices, gait with a standard walker, and gait with a wheeled walker.(CUBO et al., 2003)The authors compared walking time, the number and duration of freezing episodes, and the average duration of freezing.(CUBO et al., 2003).The authors found that walking speed was higher without the use of the devices.(CUBO et al., 2003)Furthermore, the use of standard walkers significantly increased the number and duration of freezing episodes.(CUBO et al., 2003).

Although the results of the aforementioned studies indicate that the use of walking aids may worsen gait patterns in individuals with PD, all studies investigated the immediate effects of using such devices, since individuals received instructions and were

familiarized with the devices only a few minutes before the assessment measures.(BRYANT et al., 2012; CUBO et al., 2003; KEGELMEYER et al., 2013)Thus, despite being commonly prescribed in clinical practice, the effects of training and use of assistive devices, including single-point canes, on gait speed in individuals with PD are poorly understood.

OBJECTIVES

2.1 Primary objective

The primary objective of this project will be to investigate the effect of cane training and use on gait speed in individuals with PD.

2.2 Secondary objectives:

The secondary objectives will be: to investigate the effect of training and cane use on cadence, step length, gait confidence, mobility, freezing of gait in individuals with PD, and to investigate the satisfaction of individuals with PD about the cane use.

MATERIALS AND METHODS

Outline

This is a randomized clinical trial with blinded assessors. The study will be submitted to the Research Ethics Committee of the Federal University of Minas Gerais, and the study protocol will be registered in [the relevant directory/institution].www.ClinicalTrials.govThe study will follow the Consolidated Standards of Reporting Trials (CONSORT) guidelines.(CUSCHIERI, 2019).

Participants

Individuals diagnosed with PD will be recruited from the community in the city of Belo Horizonte, Minas Gerais, Brazil. Recruitment will be done through patient lists from other projects with the same population, as well as through outreach on social media, hospitals, primary health care units, and university spaces. Individuals who meet the following criteria will be included.: age ≥ 40 years; PD diagnosis confirmed by a neurologist; Classification of II to IV in the Hoehn & Yahr (HY) Disability Stages(GOULART et al., 2005) use of anti-Parkinsonian medication and medical stability for at least 6 months; ability to walk independently in a 14-meter corridor at a walking speed equal to or less than 1.1 m/s; ability to use a single-point cane correctly and safely

while walking without prior use of assistive devices. To assess the individual's ability to use the cane, the following criteria will be adopted: 1) absence of visual, cognitive, physical, or auditory impairment that prevents training and use of the device; and 2) absence of loss of balance while walking with the device.(KEGELMEYER et al., 2013; MCCANDLESS et al., 2016)Individuals with cognitive impairment, as assessed by the Mini-Mental State Examination.(BRUCKI et al., 2003)or that present any other neurological, cardiopulmonary, or musculoskeletal condition that may compromise the performance of the tests will be excluded.

Randomization

Individuals will be randomly assigned to the experimental and control groups. The allocation ratio will be 1:1, according to a randomization plan generated by a website. The randomization sequence will be kept in sealed opaque envelopes. The envelopes will be prepared before the start of the study by a research assistant not involved in the study.

Procedures

Individual assessments will be conducted at NEUROLAB/UFMG. Assessments will be conducted by a trained researcher blinded to group allocation. All assessed individuals will be informed of the study objectives and will sign the Informed Consent Form. During the initial assessment, eligibility criteria will be verified. For individuals who meet these criteria, sociodemographic data (sex, age, education, socioeconomic level, and occupation) and clinical data (disease duration and Unified PD Rating Scale (UPDRS)) will be collected.(GOULART et al., 2005)For the UPDRS scale, section III will be specifically evaluated, as it is used to assess the impact of the disease on the individual's motor function.(SHEARIN et al., 2021; GOULART et al., 2005).

The included participants will be randomly allocated to the experimental group or control group by the physiotherapist responsible for the intervention. This physiotherapist will be blinded to the assessment performed and previously trained on the intervention procedures. After allocation, individuals in the intervention group will be instructed to maintain their usual healthcare routines and they will receive training on how to use a cane. The training will be conducted at NEUROLAB, UFMG, whose approval has already been obtained, and will be offered in four 40-minute sessions spaced 15 to 22 days apart. In addition, they will be instructed to use the cane during their daily mobility activities, both indoors and outdoors, from the first day of training. To monitor adherence

to cane use, individuals will receive a diary to record their daily use of the device during mobility activities.

Individuals allocated to the control group will be instructed to maintain their usual health care routine and not start using any walking aids during the study period. To control the amount of attention received, individuals in the control group will receive a placebo intervention containing global stretching and health education. The intervention will be carried out at NEUROLAB, UFMG, and will be offered by the same physiotherapist responsible for training the experimental group in four 40-minute sessions spaced 15 to 22 days apart.

All individuals will be reassessed after randomization by the same physiotherapist who performed the first assessment, who will be blinded to the group to which the individual was allocated. Participants will be instructed not to discuss with the assessors the group to which they were allocated. One month after the reassessment, individuals will be reassessed again (follow-up). In these two reassessments, data on the primary and secondary outcome variables will be collected. The individuals in the experimental group will continue to use the cane, but no further instructions regarding its use will be provided during this period between the initial reassessment and the follow-up reassessment. All assessments and training will be conducted during the "on" period of medication. Furthermore, the vital data from all participants will be monitored before and after each intervention session, and the presence of pain and/or fatigue will be questioned during the interventions.

If the experimental group shows improvement in the primary outcome compared to the control group, participants in the control group will be offered the opportunity to undergo cane training after the study is completed.

Intervention of the experimental group

The height of the cane will be adjusted.so that each individual's elbow is at approximately 30 degrees of flexion.(BRYANT et al., 2012)Regarding which side to use the cane on, patients will be instructed to use the device on the side they use most (dominant) or the side with the least impairment.(GLISOI et al., 2012).

The gait training protocol will be based on the Exercise Guidelines for Gait Dysfunction in PD.(NI et al., 2018)According to these guidelines, for gait-related outcomes, specific gait training should be preferred over general exercise programs.(NI et al., 2018)Furthermore, supervised training is recommended, as it is associated with

greater improvement in motor function.(NI et al., 2018)The authors further suggest that the program be initiated at a comfortable, auto-selected driving speed, progressing to maximum speeds.(NI et al., 2018)Therefore, the training will be conducted under the supervision of a physiotherapist, and the training protocol will include gait training on different surfaces and speeds, using a cane, divided into the following stages: 1) gait training on the ground at a comfortable speed, on a flat and stable surface (warm-up); 2) gait training on the ground at a comfortable speed, on a steep and stable surface (ramp); 3) gait training on the ground at maximum speed, on a flat and stable surface; 4) gait training on the ground at a comfortable speed, on a flat and stable surface (cool-down). Stages 1 and 4 will last 5 minutes each. Stages 3 and 4 will last 10 minutes each. A break will be given for the patient to rest between stages and whenever necessary. In addition, at the beginning of each training session, individuals will have the opportunity to ask any questions they may have about using the cane in their daily life context.

Control group intervention

The control group will receive a placebo intervention based on protocols from previous studies. (Avelino et al., 2018; Martins et al., 2017) The intervention will include global stretching exercises for upper and lower limbs and health education. During the intervention, 20 minutes of static global stretching will be performed, followed by 20 minutes of general health care guidance. The stretches will be performed in three sets of 30 seconds each and will involve different muscle groups. (Avelino et al., 2018; Martins et al., 2017) If the participant is unable to perform self-stretching, the researchers responsible for implementing the intervention will provide assistance. Guidance on general health care will include information on PD and fall prevention and will be based on the Portuguese version of the European Physiotherapy Guideline for PD (CAPATO et al., 2015).

Outcome measures

Primary outcome measure

Gait speed

Gait speed will be measured using the 10-meter Gait Speed Test. This will be done using a 14-meter corridor where the central 10 meters will be used to calculate gait speed. The first two meters and the last two meters will be disregarded from the assessment as

they correspond to the individual's acceleration and deceleration time during walking. The individual will be instructed to walk the indicated space first at their usual speed and then at their maximum speed. Gait speed will be calculated based on the time taken for the individual to cover the central 10 meters. This test has adequate measurement properties for evaluating this outcome in individuals with PD and is widely used in clinical practice.(LIM et al., 2005).An initial test will be conducted to familiarize the patient, followed by a second test to record the measurement. (Lindholm et al., 2018)If the individual experiences any freezing episodes during the test, the number of episodes and the freezing time will be recorded. Bloem, 2016).

Secondary outcome measures

Stride length and cadence:

For measuring step length and cadence, the number of steps taken during the 10m speed test will be counted.(CAPATO et al., 2015).To calculate average stride length, the total distance is divided by the number of steps. To calculate cadence, the number of steps is divided by the time taken to cover 10 meters, in seconds.(CAPATO et al., 2015).

Gait confidence

Confidence in gait will be assessed using the Gait Efficacy Scale-Brazil (mGES-Brazil).(AVELINO et al., 2018)The mGES-Brazil is a ten-item scale that assesses an individual's confidence during gait in challenging circumstances, such as walking on different surfaces.(AVELINO et al., 2018)Each item on the scale is individually scored on a Likert scale from zero to ten points, with higher scores indicating greater confidence.(AVELINO et al., 2018)The scale has already been cross-culturally adapted for Brazilian Portuguese and has shown adequate measurement properties for assessing this outcome.(AVELINO et al., 2018).

Functional mobility

Functional mobility will be assessed using the Timed Up and Go (TUG) test. The TUG is an easy-to-administer test that measures the time it takes an individual to stand up from a chair, walk 3 meters, turn around, and return to the chair.(CAPATO et al., 2015)This test also presents adequate measurement properties for evaluating this outcome in individuals with PD, and a TUG time > 8.5 seconds for completing the test is associated

with a higher risk of falls in individuals with PD.(CAPATO et al., 2015).To measure this outcome, an initial test will be conducted to familiarize the patient.(CAPATO et al., 2015). Immediately after the test, it will be performed again and the time of the second test will be recorded.(CAPATO et al., 2015)As with the gait speed test, the number and duration of possible episodes freezing will be recorded (Bloem, 2016).

Freezing of gait

Freezing during gait will be assessed using the Gait Freezing Questionnaire (*Freezing of gait questionnaire*, FOG-Q). This questionnaire has six items whose objective is to evaluate freezing during gait in patients with PD (Baggio et al., 2012; Oliveira, 2010).The instrument has already been cross-culturally translated into Brazilian Portuguese (Baggio et al., 2012)Furthermore, the questionnaire has adequate measurement properties and is considered reliable for screening and measuring the severity of frostbite, as well as for evaluating this outcome after interventions. (Baggio et al., 2012)In addition to the questionnaire, the number and duration of freezing episodes that patients experience during the gait speed test and the TUG will be reported.

Satisfaction with using the cane

Satisfaction with cane use will be collected from individuals in the experimental group at the end of their participation in the study. For this purpose, the Quebec User Evaluation of Satisfaction with Assistive Technology (QUEST 2.0) will be used. developed with the goal of evaluating user satisfaction with assistive technology in various aspects such as comfort, safety, weight, ease of use, etc. (Carvalho et al., 2014)The questionnaire has 12 items, scored from zero to five, where higher scores reflect greater patient satisfaction with the assistive technology. (Carvalho et al., 2014)This instrument has already been cross-culturally translated into Brazilian Portuguese and has proven reliable and valid for measuring user satisfaction with assistive walking technology.(Carvalho et al., 2014).

Sample calculation

Twenty-six individuals will be included in this study. The sample size calculation was performed considering the clinically important difference in the primary outcome measure, gait speed. (Hass et al., 2014)Considering a significance level (α) of 5% and a

power of 0.80, an n=9 was found for each group. Therefore, a sample size of 18 individuals. Assuming a sample loss of 30% of individuals throughout the study, a total sample of 26 individuals was determined, with 13 in each group.

Data analysis

The analysis will be performed by an independent researcher, blinded to the randomization of the groups, using intention-to-treat and per- protocol analysis. The normality of the data will be tested for all continuous numerical variables. Descriptive statistics will be used to characterize the sample and to investigate satisfaction with the use of the cane. The difference between the groups for the other outcome variables (gait speed, gait confidence, cadence, step length, functional mobility, freezing of gait) will be evaluated using repeated measures ANOVA, considering the measurements collected at the initial assessment, after training, and at follow-up. All statistical analyses will be performed using SPSS statistical software (SPSS Inc., Chicago, IL, United States). A significance level of α=5% will be established for all inferential analyses.

References

Aaseth J, Dusek P, Roos PM. Prevention of progression in PD. Biometals. 2018;31(5):737-747. doi:10.1007/s10534-018-0131-5

Avelino PR, Menezes KKP, Nascimento LR, et al. Cross-cultural adaptation of the Modified Gait Efficacy Scale for individuals with stroke. Rev Ter Ocup Univ Sao Paulo. 2018 ;29(3):230-6. https://doi.org/10.11606/issn.2238-6149.v29i3p230-236

Avelino PR, Nascimento LR, Menezes KKP, Scianni AA, Ada L, Teixeira-Salmela LF. Effect of the provision of a cane on walking and social participation in individuals with stroke: protocol for a randomized trial. Braz J Phys Ther. 2018;22(2):168-173. doi: 10.1016/j.bjpt.2017.11.002.

Baggio JA0, Curtarelli MB, Rodrigues GR, et al. Validity of the Brazilian Version of the Freezing of Gait Questionnaire. Archives of Neuro-Psychiatry. 2012 (70)8. doi.org/10.1590/S0004-282X2012000800008>.

Bloem BR, Marinus J, Almeida Q, Dibble L, Nieuwboer A, Post B, Ruzicka E, Goetz C, Stebbins G, Martinez-Martin P, Schrag A; Movement Disorders Society Rating Scales Committee. Measurement instruments to assess posture, gait, and balance in PD: Critique and recommendations. Mov Disord. 2016 Sep;31(9):1342-55. doi: 10.1002/mds.26572.

Bloem BR, Okun MS, Klein C. PD. Lancet. 2021;397(10291):2284-2303. doi:10.1016/S0140-6736(21)00218-X

Borghammer P. How does PD begin? Perspectives on neuroanatomical pathways, prions, and histology. Mov Disord. 2018;33(1):48-57.doi:10.1002/mds.27138

Bouça-Machado R, Maetzler W, Ferreira JJ. What is Functional Mobility Applied to PD?. J Parkinsons Dis. 2018;8(1):121-130. doi:10.3233/JPD-171233

Braak H, Del Tredici K, Rüb U, et al. Staging of brain pathology related to sporadic PD. Neurobiol Aging. 2003;24(2):197-211. doi:10.1016/s0197-4580(02)00065-9

Brucki SM, Nitrini R, Caramelli P, et al. Suggestions for the use of the Mini-Mental State Examination in Brazil. Arq Neuropsiquiatr. 2003;61(3B):777-781. doi:10.1590/s0004-282x2003000500014

Bryant MS, Pourmoghaddam A, Thrasher A. Gait changes with walking devices in persons with PD. Disabil Rehabil Assist Technol. 2012;7(2):149-152. doi:10.3109/17483107.2011.602461

Bryant MS, Rintala DH, Graham JE, Hou JG, Protas EJ. Determinants of use of a walking device in persons with PD. Arch Phys Med Rehabil. 2014;95(10):1940-1945. doi:10.1016/j.apmr.2014.06.002

Capato TTC, Domingos JMM, Almeida LRS. Portuguese Version of the European Physiotherapy Guideline for PD. Omnifarma; 2015. Available from: https://www.parkinsonnet.nl/app/uploads/sites/3/2019/11/diretriz_dp_brasil_versao_final_publicada.pdf

Carvalho KE, Gois Júnior MB, Sá KN. Translation and validation of the Quebec User Evaluation of Satisfaction with Assistive Technology (QUEST 2.0) into Brazilian Portuguese. Rev Bras Reumatol. 2014;54(4):260-267. doi:10.1016/j.rbr.2014.04.003

Coppola VS. Alpha-Synuclein Aggregation in PD: Importance of Endoplasmic Reticulum Stress. Doctoral thesis - Federal University of Paraná, Sector of Biological Sciences, Postgraduate Program in Cellular and Molecular Biology. Defense: Curitiba, 2018. Available at: https://acervodigital.ufpr.br/handle/1884/70618?show=full

Cube E, Moore CG, Leurgans S, Goetz CG. Wheeled and standard walkers in PD patients with gait freezing. Parkinsonism Relat Disord. 2003;10(1):9-14. doi:10.1016/s1353-8020(03)00060-9

Cuschieri S. The CONSORT statement. Saudi J Anaesth. 2019;13(Suppl 1):S27-S30. doi:10.4103/sja.SJA_559_18

di Biase L, Di Santo A, Caminiti ML, et al. Gait Analysis in PD: An Overview of the Most Accurate Markers for Diagnosis and Symptoms Monitoring. Sensors (Basel). 2020;20(12):3529. Published 2020 Jun 22. doi:10.3390/s20123529

GBD 2015 Neurological Disorders Collaborator Group. Global, regional, and national burden of neurological disorders during 1990-2015: a systematic analysis for the Global Burden of Disease Study 2015. Lancet Neurol. 2017;16(11):877-897. doi:10.1016/S1474-4422(17)30299-5

Glisoi SFN, Ansai JH, Silva TO, et al. Auxiliary devices for walking: guidance, demands and falls prevention in elderly people. Geriatr Gerontol Aging. 2012 6(3):261-72. Available at: https://cdn.publisher.gn1.link/ggaging.com/pdf/v6n3a06.pdf

Goulart F, Pereira LX, Goulart DF. Use of scales for the assessment of PD in Physiotherapy. Physiotherapy and Research. 2005; 11(1) doi.org/10.1590/fpusp.v11i1.76385

Hass CJ, Bishop M, Moscovich M, et al. Defining the clinically meaningful difference in gait speed in persons with Parkinson disease. Journal of Neurologic Physical Therapy. 2014;38(4):233-238. doi:10.1097/NPT.0000000000000055

Kader M, Jonasson SB, Iwarsson S, et al. Mobility device use in people with PD: A 3-year follow-up study. Acta Neurologica Scandinavica. 2018;138(1):70-77. doi:10.1111/ane.12942

Lim LIIK, van Wegen EEH, de Goede CJT, et al. Measuring gait and gait-related activities in Parkinson's patients own home environment: a reliability, responsiveness and feasibility study. Parkinsonism & Related Disorders. 2005;11(1). doi:10.1016/j.parkreldis.2004.06.003

Martins JC, Aguiar LT, Nadeau S, Scianni AA, Teixeira-Salmela LF, Faria CDCM. Efficacy of Task-Specific Training on Physical Activity Levels of People With Stroke:

Protocol for a Randomized Controlled Trial. Phys Ther. 2017 Jun 1;97(6):640-648. doi: 10.1093/physth/pzx032.

Marsili L, Rizzo G, Colosimo C. Diagnostic criteria for PD: From James Parkinson to the concept of prodromal disease. Frontiers in Neurology. 2018;9(MAR). doi:10.3389/fneur.2018.00156

McCandless PJ, Evans BJ, Janssen J, Selfe J, Churchill A, Richards J. Effect of three cueing devices for people with PD with gait initiation difficulties. Gait Posture. 2016;44:7-11. doi:10.1016/j.gaitpost.2015.11.006

Ni M, Hazzard JB, Signorile JF, Luca C. Exercise Guidelines for Gait Function in PD: A Systematic Review and Meta-analysis. Neurorehabilitation and Neural Repair. 2018;32(10):872-886. doi:10.1177/1545968318801558

Oliveira JA. Validation of the Brazilian version of the balance and gait scale (GABS) and analysis of fall risk in individuals with PD and healthy subjects. 2010. 10.11606/D.17.2010.tde-18122014-101553

Poewe W, Espay AJ. Long duration response in PD: Levodopa revisited. Brain. 2020;143(8):2332-2335. doi:10.1093/brain/awaa226

Ray Dorsey E, Elbaz A, Nichols E, et al. Global, regional, and national burden of PD, 1990–2016: a systematic analysis for the Global Burden of Disease Study 2016. The Lancet Neurology. 2018;17(11):939-953. doi:10.1016/S1474-4422(18)30295-3

Raza C, Anjum R, Shakeel N ul A. PD: Mechanisms, translational models and management strategies. Life Sciences. 2019;226:77-90. doi:10.1016/j.lfs.2019.03.057

Schapira AHV, Chaudhuri KR, Jenner P. Non-motor characteristics of Parkinson disease. Nature Reviews Neuroscience. 2017;18(7):435-450. doi:10.1038/nrn.2017.62

Shearin S, Medley A, Trudelle-Jackson E, Swank C, Querry R. Differences in predictors for gait speed and gait endurance in PD. Gait Posture. 2021;87:49-53. doi:10.1016/j.gaitpost.2021.04.019

Tomlinson CL, Herd CP, Clarke CE, et al. Physiotherapy for PD: A comparison of techniques. Cochrane Database of Systematic Reviews. 2014;2014(6):1-119. doi:10.1002/14651858.CD002815.pub2

FINANCIAL BUDGET

**Materials**	**Unit cost**	**Total cost**
4 packs of A4 size office paper	R$26.99	R$107.96
4 printer cartridges	R$90.00	R$360.00
13 Single-point canes	R$50.00	R$650.00
Total: R$ 1117.96		

All expenses for this study will be covered by the researchers.

TIMELINE

Period	2023/2	2024/1	2024/2	2025/1	2025/2	2026/1	2026/2
Bibliographic update	■	■	■	■	■	■	■
Fulfillment of credits	■	■	■	■			
Submission of the project to the Ethics Committee	■	■	■				
Submission of the project to funding agencies.	■	■	■				
Submission of the protocol for publication.			■	■			
Recruitment period				■	■	■	
Initial assessment				■	■	■	
Revaluation				■	■	■	
Follow-up				■	■	■	
Partial and final statistical analysis and interpretation of results.					■	■	
Thesis preparation and defense						■	■
Forwarding for publication							■

Note: Recruitment and data collection will only begin after approval from the Ethics Committee. Therefore, adjustments to this timeline may be made depending on the date of the Ethics Committee approval.

APPENDIX 1 - FREE AND INFORMED CONSENT FORM

FREE AND INFORMED CONSENT FORM

FREE AND INFORMED CONSENT FORM No.____________________

PROJECT TITLERESEARCH STUDIES: EFFECT OF TRAINING AND USE OF A CANE ON THE MOBILITY OF INDIVIDUALS WITH PD: A RANDOMIZED CLINICAL TRIAL

INVESTIGATORS:

- Prof. Christina Danielli Coelho de Morais Faria, physiotherapist, Ph.D. Professor in the Department of Physiotherapy at the Federal University of Minas Gerais (UFMG). Telephone: (31) 3409-7448; (31) 3409-4783;cdcmf@ufmg.br
- Jordana de Paula Magalhães, physiotherapist, student of the Postgraduate Program in Rehabilitation Sciences at UFMG. Telephone: (31) 987733602;jordanamagalhaes.jpm@gmail.com

INFORMATION:

You are invited to participate in a research project entitled: "Effect of cane training and use on the mobility of individuals with PD: a randomized clinical trial" to be developed by the Department of Physiotherapy of the School of Physical Education, Physiotherapy and Occupational Therapy of the Federal University of Minas Gerais. Although commonly used by individuals with PD, the effect of cane training and use on the mobility of these patients is unknown. Therefore, the objective of this research project is to investigate the effect of cane training and use on gait speed, gait confidence, cadence, step length, mobility, and freezing of gait in individuals with PD, and to investigate the individuals' satisfaction with the use of the device.

DESCRIPTION OF THE TESTS AND INTERVENTIONS TO BE PERFORMED

Initial assessment

If you agree to participate, you will initially undergo an interview to collect your personal data, followed by a physical examination, both administered by a previously trained examiner. This will involve a pre-structured questionnaire that will include questions about you and your health condition. In addition, you will perform some tests commonly used in clinical practice to obtain information about your gait speed, gait confidence, cadence, step length, mobility, and freezing during gait. Your safety, well-being, and comfort will be considered throughout all procedures. The examiners will respect measures to prevent the spread of infectious diseases, such as the use of disposable surgical masks and the availability of 70% alcohol-based hand sanitizer.

Study groups

Upon entering this research project, you will be randomly assigned to one of the following groups: 1) Intervention group: Individuals in the intervention group will receive a single-tip cane that must be used during all their locomotion activities for 22 days. Individuals will receive four training sessions on how to use the cane during this period. During the training sessions, individuals can ask any questions about using the device and practice its use in different gait activities. The training sessions will last 40 minutes and will be administered by a trained physiotherapist. In addition, individuals will receive a diary to record their daily use of the cane during locomotion activities. 2) Control group: The intervention for the control group consists of global stretching and health education. The intervention will be offered in four 40-minute sessions spaced 15 to 22 days apart and will be administered by a trained physiotherapist. Both groups should maintain their usual health care routines. If the experimental group shows improvement at the end of the study, participants in the control group will be offered the opportunity to undergo cane training as performed by the experimental group.

Procedures

Initially, an initial assessment will be conducted where several measures will be evaluated, and you will answer some questionnaires and perform gait and mobility assessment tests. Following this, you will be allocated to one of the groups and receive instructions relevant to your group allocation. The same procedures from the initial assessment, i.e., all tests and measurements employed, will be repeated 22 days after the first assessment and at a one-month follow-up after the second assessment. All procedures, tests, measurements, and interventions to be performed in this study are standardized and commonly adopted in clinical practice or in previously conducted scientific studies. Your safety, well-being, and comfort will be considered throughout all procedures.

Risks

The risks of participating in this study include muscle soreness during and after the tests, as these may require greater physical exertion than individuals perform daily. If this occurs, participants will be assisted by the responsible researcher for the necessary time using recommended procedures. In addition, participants may feel tired and fatigued during the assessments or training. To minimize this discomfort, a rest period will be provided between tests and during training. Any discomfort experienced by the participant should be reported so that the researchers can take appropriate measures to minimize it.

Benefits

You and future patients may benefit from the results of this study, especially since its main objective is to investigate the effects of using a device commonly used by individuals with PD.

Based on the information obtained in this study, it will be possible to understand the effects of cane use on gait speed, gait confidence, cadence and step length, mobility, and freezing of gait in these individuals. Furthermore, this study will also gather information about the satisfaction with cane use among patients in the experimental group.

Confidentiality

To guarantee your anonymity and the confidentiality of your data, this study will use a numerical password for your identification. This password will be used in all your tests; your name will not be used. If information originating from this study is published in a scientific journal or event, you will not be individually identified, as you will be represented by the password. The data collected in this study will be stored for a period of ten years.

Voluntary nature of the study and payment.

Your participation in this study is entirely voluntary, and you are free to agree or not to participate. If you wish, you may withdraw from the study at any time without any personal loss to you. Participation in the study will not incur any costs for you, and there will be no payment for your participation. The tests and all

materials used in the research, including the single-tip cane, will be free of charge. If necessary, any additional transportation costs will be the responsibility of the researchers.
After reading the information above, if you wish to participate, please complete and sign this document in duplicate, filling out the spaces provided on every page. One copy will remain with the participant and the other with the researcher.

DECLARATION AND SIGNATURE

I,____I have read and understood all the information provided about the study, and the objectives, procedures, and technical language were satisfactorily explained. I have had sufficient time to consider the above information and have had the opportunity to ask all my questions. I am voluntarily signing two copies of this document, one for myself and one for the researchers, and I have the right to discuss any questions I may have regarding the research with:

- Christina Danielli Coelho de Morais Faria, physiotherapist, Ph.D. Professor in the Department of Physiotherapy at the Federal University of Minas Gerais (UFMG). Telephone: (31) 3409-7448; (31) 3409-4783;cdcmf@ufmg.br
- Jordana de Paula Magalhães, physiotherapist, student of the Postgraduate Program in Rehabilitation Sciences at UFMG. Telephone: (31) 987733602;jordanamagalhaes.jpm@gmail.com

Furthermore, in case of ethical questions you have the right to contact: UFMG Research Ethics Committee: (31) 3409-4592. Address: Av. Antônio Carlos, 6627 Unidade Administrativa II, sala 2005. Campus Pampulha, BH/MG. CEP 31270-901.

By signing this consent form, I am indicating that I agree to participate in this study.

____ _______________________

Participant Signature Datthe
RG:__________________________CPF:____________________________________
End.:____
____

____ _______________________

Signature of the Principal Investigator Datthe
Christina Danielli Coelho de Morais Faria
Jordana de Paula Magalhães

APPENDIX II - EVALUATION FORM

RESEARCH PROJECT:
**EFFECT OF TRAINING AND USE OF A CANE ON THE MOBILITY OF INDIVIDUALS WITH PARKINSON'S DISEASE: A RANDOMIZED CLINICAL TRIAL**

EVALUATION FORM (initial, re-evaluation or follow-up):________________________

DATE:___________________TIME:_____________________CODE:______________________

1. DEMOGRAPHIC DATA

1.1 Name:____
1.2 Address: ____
1.2 Telephone:____ Gender: ( ) Female ( ) Male
1.3 Date of Birth:______________________________ Age:_________________________
1.4 Marital status:_____________________ Lives with:_____________________________________
1.5 Education (years studied):___________________ Training:_________________________
1.6 Occupation: ____
1.7 Companion (name and contact):____

2 Clinical Data on PD

2.1 Time since onset of symptoms:______________Diagnosis time:_______________
2.2 Neurologist Diagnosis: ( ) YES ( ) NO
2.3 The more committed side:_____________Use of deep stimulation: ( ) YES ( ) NO
2.4 HY:______________UPDRS-III:____________

3 GENERAL CLINICAL DATA

3.1 Dominant MS:_____________Dominant MI: _______________
3.2 Visual impairment: ( ) No ( ) Yes   Deficit Hearing: ( ) No ( ) Yes
3.3 Walks independently: ( ) No ( ) Yes
3.4 Number of associated diseases (description):____
____

3.5 Falls: How many times have you fallen in the last week? Month? In the last 6 months?
3.6 Are you currently doing any physical exercise? ( ) No ( ) Yes Which one? _________________
How many times a week? ________________. What is the duration (in minutes)?_____________________
3.7 Are you currently undergoing any rehabilitation treatment? (e.g., physiotherapy, occupational therapy, speech therapy, etc.) ( ) No ( ) Yes Which one? ______________________
How many times a week? ________________ How long (in minutes): ____________________
3.8 Medications in use

Medication	Dose	Time	Usage time

Weight (kg): ____________ Height (cm): _______________
PA: _______________ FC: ________________ FR: ______________ SatO2: _________________

Able to use a single-tip cane: ( ) Yes ( ) No Reason: ________________________

Mini Mental State Examination

Time orientation		Points	Score
**What day is it today?**		1	
**What month is it?**		1	
**What year is it?**		1	
**What day of the week is it?**		1	
**What is the approximate time?**	Consider a variation of one hour or less.	1	
**Spatial orientation**		Points	Score
**Where are we located?**	Consulting room, bedroom, living room - pointing to the floor	1	
**What place is this?**	Pointing around in a broader sense: hospital, nursing home, one's own home.	1	
**What neighborhood are we in, or what is the name of a nearby street?**		1	
**Which city are we in?**		1	
**What state are we in?**		1	
**Immediate memory**		Points	Score
**I'm going to say three words and you will repeat them afterward: car, vase, brick.**	Of 1 point to each word repeatedcorrectly the first time, although they can be repeated. Up to three times for learning purposes, if there are mistakes.	3	
**CALCULATION**		Points	Score
Subtracting sevens sequentially: **How much is it? 100-7, 93-7, 86-7, 79-7, 72-7, 65**	Award 1 point for each correct answer. If there is an error, correct it and proceed. Consider it correct if the examinee spontaneously self-corrects. **(SEE*)**	5	
**Evocation of words**		Points	Score
**What words did you just say? repeat?**	*Ask what words the subject had just used. Repeat - 1 point for each*	3	
**Appointment**		Points	Score
**What object is this?**	Ask the subject to name the objects shown (clock, pen) - 1 point for each.	2	
**Repetition**		Points	Score
**Listen carefully: I'm going to tell you a sentence. And I want you to repeat after me: "Neither here, nor there, nor anywhere."**	Consider only if the repetition is perfect (1 point)	1	
**Command**		Points	Score
**"Take this paper with your right hand." (1 point), fold it in half (1 point) and place it on the floor (1 point)".**	Total of 3 points. If the subject asks for help in the middle of the task, do not give hints.	3	
**Reading**	Show the individual the written phrase 'CLOSE YOUR EYES' and ask them to do as instructed. Do not assist if you ask for help or if you only read. The phrase without carrying out the command.	1	
**Phrase** Write a sentence.	Ask the individual to write a sentence. If you don't understand the meaning, help with: a sentence that has a beginning, middle, and end; Something that happened today; something you want to say. Grammatical or spelling errors are not considered for grading purposes.	1	
**Copy of the drawing:** Make aCopy this drawing as best as possible.	Show the model and ask them to do their best. Only consider if there are 2 intersecting pentagons (10 angles) forming a figure with four sides or with two angles (1 point).	1	
TOTAL	30		

CUTTING POINTS

13 for illiterate people

18 for low/medium education (1 to 7 years of schooling)

26 for higher education (8 or more years of schooling)

Spell the word WORLD backwards.*one point for each letter in the positionCorrect - *Note: Only the grade*

for the best performance will be considered.

UNIFIED PD RATE SCALE (UPDRS) (PARTIAL)

III- MOTOR EXPLORATION

1. Spoken language
0 = Normal.
1 = Slight loss of expression, diction, and/or voice volume.
2 = Monotonous, drawn-out, but understandable; moderate change.
3 = Change marked, difficult to understand.
4 = Unintelligible

2. Facial expression
0 = Normal
1 = Minimal hyponymy; could be normal ("poker player face")
2 = Slight but clearly abnormal decrease in facial expression.
3 = Moderate hyponymia; lips parted on some occasions.
4 = Fixed or mask-like face with severe or total loss of facial expression, lips parted ≥ 0.6 cm)

3. Resting tremor
0 = Absent
1=Mild and infrequent
2 = Of small amplitude and continuous, or of moderate amplitude and intermittent occurrence.
3 = Of moderate amplitude and present almost continuously.
4 = Of marked amplitude and present almost continuously.

4. Action or postural tremor of the hands:
0 = Absent
1=Light; present during the activity
2 = Of moderate amplitude, present during activity.
3=Moderate amplitude, present when maintaining a posture as well as during activity.
4 = Of marked amplitude, it makes feeding difficult.

5. Stiffness: (Assessed through passive mobilization of the major joints, with the patient seated and relaxed. Do not assess the cogwheel rigidity phenomenon).
0 = Absent
1=Slight sensation only perceived when activated by contralateral movements or other movements.
2 = Mild to moderate.
3 = Marked, but allows easy access to the maximum range of motion.
4 = Severe, the maximum range of motion is achieved with difficulty.

6. Digital dexterity. (The patient taps their thumb against their index finger rapidly and successively with the greatest possible amplitude; each hand separately).
0 = Normal
1 = Slightly slow and/or reduced amplitude.
2 = Moderate alteration. Clear and early fatigue. Movement may occasionally stop.
3 = Serious alteration. Frequent hesitation when initiating movement or pauses while performing the movement.
4 = You can only perform the exercise.

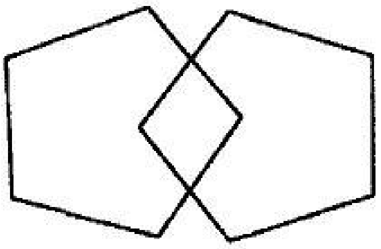

7. Hand movements. (The patient opens and closes their hand rapidly and successively with the greatest possible amplitude; each hand separately).
0 = Normal
l = Slight slowness and/or reduction in amplitude.
2 = Moderate alteration. Clear and early fatigue. Movement may occasionally stop.
3 = Serious alteration. Frequent indecision in initiating movement or pauses while performing the movement.
4 = You can only perform the exercise.

8. Rapid, alternating hand movements: (Pronation-supination movements, vertically or horizontally, with the greatest possible range of motion and both hands simultaneously).
0 = Normal
l = Mild slowness and/or reduction in amplitude
2 = Moderate alteration. Clear and early fatigue. Movement may occasionally stop.
3 = Serious alteration. Frequent hesitation when initiating movement or pauses while performing the movement.
4 = You can only perform the exercise.

9. Leg agility: (The patient taps their heel against the ground in rapid succession, lifting the leg completely. The range of motion should be 7 to 8 cm.)
0 = Normal
1 = Slight slowness and/or reduction in amplitude.
2 = Moderate alteration. Clear and early fatigue. Movement may occasionally stop.
3 = Serious alteration. Frequent hesitation when initiating movement or pauses while performing the movement.
4 = You can only perform the exercise.

10. Getting up from a chair. (The patient attempts to stand up from a wooden or metal chair with a vertical backrest, keeping their arms crossed over their chest.)
0 = Normal
1 = Slow or requires more than one attempt.
2 = Stand up using the arms of the chair for support.
3. Tends to fall backward and may try several times even if able to get up without help.
4 = Cannot get up from the chair without help.

11. Posture
0 = Erected normally.
1 = Not fully erect, slightly bent, can be normal in elderly people.
2 = Moderately stooped posture, clearly abnormal, may be leaning slightly to one side.
3 = Intensely stooped posture with kyphosis; may be moderately inclined to one side.
4 = Marked flexion with extreme postural alteration

12. March
0 = Normal
1. The gait is slow, the feet may drag, and the steps may be short, but there is no propulsion or festination.
2 = Walks with difficulty, but needs little or no help; there may be some festination, short steps, or propulsion.
3 = Severe gait disorder requiring assistance.
4. Marching is impossible, even with assistance.

13. Postural stability (Observe the response to a sudden backward displacement, caused by a push on the shoulders, with the patient standing with eyes open and feet slightly apart. Warn the patient beforehand)
0 = Normal
1=Retropulsion, although recovery occurs without assistance.
2 = Absence of postural reflex; could have fallen if the evaluator had not prevented it.
3 = Very unstable; tendency to lose balance spontaneously.
4 = Unable to stand without assistance.

14. Bradykinesia and hypokinesia. (Combination of slowness, indecisiveness, decreased arm swing, reduced range of motion, and general scarcity of movement).
0 = Absent
1 = Minimal slowness, giving the movement a decisive character; this could be normal in some people. Amplitude possibly reduced.
2 = Mild degree of slowness and scarcity of movements; clearly abnormal. There may be a decrease in amplitude.
3 = Moderate slowness, poor movement, or reduced range of motion.
4 = Marked slowness and poverty of movement with reduced amplitude of movements.

**- Hoehn and Yahr Disability Stages Scale (modified):**
0: Absence of signs of the disease
1.0: Unilateral change
1.5: Unilateral alteration with axial involvement
2.0: Bilateral alteration, without balance deficit.
2.5: Mild bilateral alteration with recovery in the push test.
3.0: Mild to moderate bilateral alteration, some postural instability, physically independent.
4.0: Severe disability, still able to walk or stand without assistance.
5.0: Confined to bed or wheelchair unless assisted.

H&Y Score:___________________________

Walking speed test without a cane(A measure after familiarization)

**Test order:**	**Time (s)**	**Speed (m/s)**
Automatically selected gear speed		
Maximum driving speed		

Freezing episodes (complete cessation of movement followed by resumption)( )No ( ) Yes Number of episodes: ______________
Freezing time: ________

Number of stumbles (loss of balance with unassisted recovery): _______

Walking speed test with a cane(A measure after familiarization)

Test order:	Time (s)	Speed (m/s)
Automatically selected gear speed		
Maximum driving speed		

Freezing episodes (complete cessation of movement followed by resumption)( )No ( ) Yes Number of episodes: ______________
Freezing time: ________

Number of stumbles (loss of balance with unassisted recovery): _______

Timed up and Go test - TUG without cane(a repetition after familiarization)
Test order:______
Time:___________________________________ Mon
Number of freezing episodes: _____________
Freezing time:
Number of stumbles: ____________________

Timed up and Go- TUG test with cane(a repetition after familiarization)
Test order:_____________
Time:___________________________________ Mon
Number of freezing episodes: _____________
Freezing time:
Number of stumbles: ____________________

Self-perception of health

In general, would you say your health is:

Excellent.. 1
Very good... 2
Good.. 3
Bad... 4
Very Bad.. 5

Compared to a year ago, how would you rate your overall health now?

Much better now than a year ago............. 1
A little better now than a year ago..... 2
Almost the same as a year ago........ 3
A little worse now than a year ago.......... 4
Much worse now than a year ago...................5

"Compared to other people your age, how would you say your health is:"

1. ( ) Better 2. ( ) Same 3. ( ) Worse

FREEZE OF GEAR SCALE (FOGQ)

1. **During your worst state you walk:**
0 Normally
1 Almost normally – a little slow.
2 Slow but completely independent
3 Do you need help or a walking aid?
4 Unable to walk

2. **Are your difficulties walking affecting your activities of daily living or your independence?**
0 Not at all
1 A little
2 Moderately
3 Severely
4 Unable to walk

3. **Do you feel like your feet are stuck to the ground while you walk, turn, or when you try to start walking (freezing)?**
0 Never
1 Very rarely – once a month
2 Rarely – once a week
3. Frequently – once a day
4 Always – every time you walk

4. **How long does your worst freezing episode last?**
0 It never happened
1 to 2 seconds
2 3 to 10 seconds
3 11 to 30 seconds
4 Unable to walk for more than 30 seconds.

5. **How long does your typical episode of hesitation to start walking (freezing) last?**
to take the first step)?
0 Nothing
1 It takes more than 1 second to start walking.
2 It takes more than 3 seconds to start walking.
3. It takes more than 10 seconds to start walking.
4. It takes more than 30 seconds to start walking.

6. **How long does your typical hesitation to turn last (freezing while turning)?**
0 Nothing
1 Around 1 to 2 seconds
2 Around 3 to 10 seconds
3 Around 11 to 30 seconds
4 Unable to complete the turn for more than 30 seconds.

Modified Gait Efficacy Scale (mGES-Brazil)

1- How confident are you that you would be able to walk safely on a flat surface, such as a wooden floor?

1	2	3	4	5	6	7	8	9	10

2- How confident are you that you would be able to walk safely on the grass?

1	2	3	4	5	6	7	8	9	10

3- How confident are you that you would be able to safely overcome an obstacle in your path?

1	2	3	4	5	6	7	8	9	10

4- How confident are you that you would be able to safely step down from a curb?

1	2	3	4	5	6	7	8	9	10

5- How confident are you that you would be able to safely climb onto a curb?

1	2	3	4	5	6	7	8	9	10

6- How confident are you that you would be able to climb stairs safely if you were holding onto a handrail?

1	2	3	4	5	6	7	8	9	10

7 How much How confident are you that you would be able to safely descend stairs if you were holding onto a handrail?

1	2	3	4	5	6	7	8	9	10

8- How confident are you that you would be able to climb stairs safely if you were NOT holding onto a handrail?

1	2	3	4	5	6	7	8	9	10

9- How confident are you that you would be able to safely descend stairs if you were NOT holding onto a handrail?

1	2	3	4	5	6	7	8	9	10

10- How confident are you that you would be able to safely walk a long distance, such as 800 meters (eight blocks)?approximately)?

1	2	3	4	5	6	7	8	9	10

No trust Total trust

SCORETOTAL

SIX-MINUTE WALK TEST WITHOUT A CANE (6MWT):

TEST ORDER: _______

**Before**	**After**
Initial blood pressure (mmHg):________	Final blood pressure (mmHg):________
Initial heart rate (bpm):_______	Final heart rate (bpm):_______
Initial FR (rpm):__________________	Final FR (rpm):_________________
Initial SpO2 (%):__________________	Final SpO2 (%):_________________
Lower limb fatigue (Borg):_____________	Lower limb fatigue (Borg):__________
Dyspnea (Borg):__________________	Dyspnea (Borg):__________________

Test	Heart rate (bpm)	SpO2 (%)	Dyspnea (Borg)	Lower limb fatigue (borg)	Distance (m)
1st minute					Not applicable
2nd minute					
3rd minute					Not applicable
4th minute					Not applicable
[illegible]					Not applicable
[illegible]					

Did it stop/pause before six minutes? ( ) No ( ) Yes
Reason:________________How long?
____
Other symptoms:________
Distance in six minutes (m):_________
Distance in two minutes (m): _________

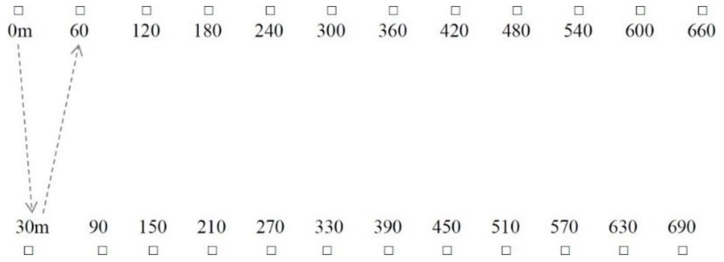

SIX-MINUTE WALK TEST WITH A CANE (6MWT):
TEST ORDER: _______

**Before**	**After**
Initial blood pressure (mmHg):________	Final blood pressure (mmHg):________
Initial heart rate (bpm):_______	Final heart rate (bpm):_______
Initial FR (rpm):__________________	Final FR (rpm):_________________
Initial SpO2 (%):__________________	Final SpO2 (%):_________________
Lower limb fatigue (Borg):_____________	Lower limb fatigue (Borg):__________
Dyspnea (Borg):__________________	Dyspnea (Borg):__________________

Test	Heart rate (bpm)	SpO2 (%)	Dyspnea (Borg)	Lower limb fatigue (borg)	Distance (m)
1st minute					Not applicable
2nd minute					
3rd minute					Not applicable
4th minute					Not applicable
[illegible]					

Did it stop/pause before six minutes? ( ) No ( ) Yes
Reason:________________How long?
____
Other symptoms:________
Distance in six minutes (m):_________
Distance in two minutes (m): _________

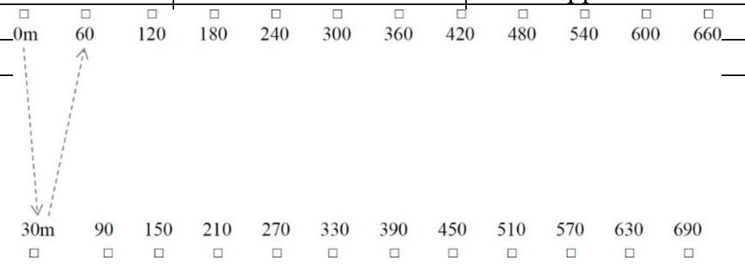

PDQ-39 - PD Questionnaire

Given that you have PD, how often have you experienced the following symptoms during the last month?

Check one box for each question.

	Never	Sometimes	Sometim es	Frequen- fearful	Always/ isimpossible for me
1. Have you had difficulty participating in recreational activities that you enjoy doing?					
2. Did you have difficulties taking care of your home (e.g., doing minor repairs, housework, cooking)?					
3. Did you have difficulty carrying shopping bags?					
4. Did you have trouble walking one kilometer (10 blocks)?					
5. Did you have trouble walking 100 meters (1 block)?					
6. Did you have trouble moving around the house as easily as you would have liked?					
7. Did you have difficulty moving around in public places?					
8. Did you need someone to accompany you when you went out?					
9. Did you feel scared or worried about falling in public?					
10. Did you stay home more than you would have liked?					
11. Did you have difficulty washing yourself?					
12. Did you have difficulty getting dressed?					
13. Did you have difficulty buttoning clothes or tying shoes?					
14. Did you have trouble writing legibly?					
15. Did you have difficulty cutting the food?					
16. Have you ever had trouble holding a drink without spilling it?					
17. Have you felt depressed?					
18. Did you feel isolated/alone?					
19. Did you feel like you could easily start crying?					
20. Did you feel angry or bitter?					
21. Did you feel anxious?					
22. Did you feel worried about your future?					
23. Was it necessary to hide your PD from other people?					
24. Did you avoid situations where you had to eat or drink in public?					

25. Have you ever felt embarrassed in public because you have PD?					
26. Were you concerned about other people's reactions?					
27. Have you had relationship problems with the people closest to you?					
Did you lack the support you needed from your spouse or partner? If you do not have a spouse or partner, check here ( )					
29. Did you lack the support you needed from your family or friends?					
30. Did you fall asleep unexpectedly during the day?					
31. Have you had trouble concentrating, for example, when reading or watching television?					
32. Did you feel that your memory was failing?					
33. Have you had disturbing dreams or hallucinations?					
34. Did you have difficulty speaking?					
35. Have you ever felt unable to communicate clearly with people?					
36. Have you felt ignored by other people?					
37. Did you experience painful muscle cramps or spasms?					
38. Did you experience pain in your joints or other parts of your body?					
39. Did you feel uncomfortably hot or cold?					

Score for each dimension
Each dimension is calculated on a scale of 0 to 100:
0 = no problem; 100 = maximum problem level
Formula for scoring each dimension
Sum of the scores for each question in dimension x 100
4 (max. score per question) x number of questions in the dimension
Dimensions
Mobility: 10 questions – 1 to 10
(scores for questions 1+2+3+4+5+6+7+8+9+10) / (4 x 10) x 100
Activities of daily living (ADLs):
(scores from questions 11+12+13+14+15+16) / (4 x 6) x 100
Emotional well-being:6 questions – 17 to 22
(scores from questions 17+18+19+20+21+22) / (4 x 6) x 100
Stigma:4 questions – 23 to 26
(scores from questions 23+24+25+26) / (4 x 4) x 100
Social Support: 3 questions – 27 to 29
(scores from questions 27+28+29) / (4 x 3) x 100
Note: If the participant indicates that they do not have a spouse/partner, question 28 can be calculated as follows:
Social support: (scores from questions 27+29) / (4 x 2) x 100
Cognition:4 questions – 30 to 33
(scores from questions 30+31+32+33) / (4 x 4) x 100
Communication: 3 questions – 34 to 36
(scores from questions 34+35+36) / (4 x 3) x 100
Body discomfort:3 questions – 37 to 39

(scores from questions 37+38+39) / (4 x 3) x 100

EVALUATION OF USER SATISFACTION WITH QUEBEC B-QUEST ASSISTIVE TECHNOLOGY (2.0)

For each of the 12 items, rate your satisfaction with the assistive technology resource and related services you experienced, using the following scale of 1 to 5:

1	2	3	4	5
Dissatisfied	Not very satisfied	More or less satisfied	Quite satisfied	Completely satisfied

If there is any item with which you are not "completely satisfied," please comment in the comments section.

1	2	3	4	5		
Insatisfeito	Pouco satisfeito	Mais ou menos satisfeito	Bastante satisfeito	Totalmente satisfeito		
RECURSO DE TECNOLOGIA ASSISTIVA<br>Qual é o seu grau de satisfação com:						
1.as dimensões (tamanho, altura, comprimento, largura) do seu recurso de tecnologia assistiva?						
Comentários:		1	2	3	4	5
2.o peso do seu recurso de tecnologia assistiva?						
Comentários:		1	2	3	4	5
3.a facilidade de ajustar (fixar, afivelar) as partes do seu recurso de tecnologia assistiva?						
Comentários:		1	2	3	4	5
4.a estabilidade e a segurança do seu recurso de tecnologia assistiva?						
Comentários:		1	2	3	4	5
5.a durabilidade (força e resistência ao desgaste) do seu recurso de tecnologia assistiva?						
Comentários:		1	2	3	4	5
6.a facilidade de uso do seu recurso de tecnologia assistiva?						
Comentários:		1	2	3	4	5
7.o conforto do seu recurso de tecnologia assistiva?						
[illegible]		[illegible]	[illegible]	[illegible]	[illegible]	[illegible]

1	2	3	4	5		
Dissatisfied	Little Fullysatisfied satisfied	More or satisfied satisfied	Quite a lot	less		
Assistive Technology Resource<br>How satisfied are you with: (continued)						
How effective is your assistive technology resource (how well does your resource meet your needs)?						
Comments:		1	2	3	4	5
SERVICES<br>How satisfied are you with:						
9.What was the delivery process (procedures, waiting time) by which you obtained your assistive technology device?						
Comments:		1	2	3	4	5
10. What about the repairs and technical assistance (maintenance) provided for your assistive technology device?						
Comments:		1	2	3	4	5
11. How would you rate the quality of professional services (information, attention) you received from using your assistive technology?						
Comments:		1	2	3	4	5
12. What follow-up services (ongoing support services) have you received for your assistive technology device?						
[illegible]		[illegible]	[illegible]	[illegible]	[illegible]	[illegible]

Below is a list with the same 12 satisfaction items. CHOOSE the 3 ITEMS that you consider the most important. Mark an X next to the 3 options of your choice.

1) Dimensions □
2) Weight □
3) Adjustments □
4) Security □
5) Durability □
6) Ease of use □
7) Comfort □
8) Effectiveness □
9) Delivery□
10) Repairs/Technical Assistance □
11) Professional services □
12) Follow-up services □

· Number of invalid responses ____

· Resource subtotal score _________

For items 1 through 8, add the scores of the valid responses and divide that sum by the number of valid items on this scale.

· Service Subtotal Score

For items 9 through 12, add the scores of the valid responses and divide that sum by the number of valid items on this scale.

· TotalQUEST _________________

For items 1 through 12, add the scores of the valid answers and divide this sum by the number of valid items.

· The three most important factors in satisfaction:

Logbook of cane use during training.

This diary should be filled out every day. In it, you should mark with an "x" all the environments in which you used the cane to get around (walk).

Check if you used a cane to get around in these situations.

DAYS	01	02	03	04	05	06	07	08	09	10	11	12	13	14	15	16	17	18	19	20
Inside your house (bedrooms, living room, etc.)																				
In the garden, backyard, or balcony of your house.																				
To walk on the street near your house.																				
To travel to places far from your home.																				
To go up or down stairs and/or ramps.																				

Check if you've experienced any of these situations.

DAYS	01	02	03	04	05	06	07	08	09	10	11	12	13	14	15	16	17	18	19	20
Inside your house (bedrooms, living room, etc.)																				
In the garden, backyard, or balcony of your house.																				
To walk on the street near your house.																				
To travel to places far from your home.																				
To go up or down stairs and/or ramps.																				
Inside your house (bedrooms, living room, etc.)																				
**Training days**																				

Start of training:____________ End of training:______________

Walking stick usage diary

This diary should be filled out every day. In it, you should mark with an "x" all the places where you used your cane to get around.

Check if you used a cane to get around in these situati

DAYS	01	02	03	04	05	06	07	08	09	10	11	12	13	14	15	16	17	18	19	20	21	22	23	24	25	26	27	28	29	30
Inside your house (bedrooms, living room, etc.)																														
In the garden, backyard, or balcony of your house.																														
For walking on the street, near your house.																														
To travel to places far from your home.																														
To go up or down stairs and/or ramps.																														

Check if you've experienced any of these situations.

DAYS	01	02	03	04	05	06	07	08	09	10	11	12	13	14	15	16	17	18	19	20	21	22	23	24	25	26	27	28	29	30
Inside your house (bedrooms, living room, etc.)																														
In the garden, backyard, or balcony of your house.																														
For walking on the street, near your house.																														
To travel to places far from your home.																														
To go up or down stairs and/or ramps.																														
