# Supplementary material for: Effect of a 3-week program of cane training and use on gait of individuals with Parkinson’s disease: Protocol for a randomized controlled trial
Source: PLoS One. 2026 Apr 16;21(4):e0341248. doi: 10.1371/journal.pone.0341248 (PMC13086423; doi:10.1371/journal.pone.0341248)
Supplement: S2 File — (PDF) [file pone.0341248.s002.pdf]

EFEITO DO TREINAMENTO E USO DA BENGALA NA MOBILIDADE DE INDIVÍDUOS COM DOENÇA DE PARKINSON: UM ENSAIO CLÍNICO ALEATORIZADO

Pesquisadora coordenadora: Christina Danielli Coelho de Morais Faria, Ph.D., professora associada do Departamento de Fisioterapia da Universidade Federal de Minas Gerais (UFMG).

Pesquisadora colaboradora: Jordana de Paula Magalhães, M.Sc., discente do Programa de Pós-Graduação em Ciências da Reabilitação, nível doutorado, UFMG.

Área de conhecimento: Ciências da Saúde

Escola de Educação Física, Fisioterapia e Terapia Ocupacional
Universidade Federal de Minas Gerais
2023

EQUIPE DO PROJETO DE PESQUISA

1. Christina Danielli Coelho de Morais Faria (Brasil). Fisioterapeuta, PhD, professora associada, em regime de dedicação exclusiva, do Departamento de Fisioterapia na Escola de Educação Física, Fisioterapia e Terapia Ocupacional (EEFFTO) da Universidade Federal de Minas Gerais (UFMG). Credenciada ao Programa de Pós-Graduação em Ciências da Reabilitação (PPGCR) da UFMG.

2. Jordana de Paula Magalhães (Brasil). Fisioterapeuta, MSc, doutoranda no PPGCR da UFMG sob orientação da professora Christina Faria (UFMG) cuja tese de doutorado será desenvolvida com os resultados deste projeto.

INTRODUÇÃO

A doença de Parkinson (DP) é uma condição clínica neurodegenerativa, progressiva e de crescente prevalência em todo o mundo(AASETH et al., 2018; BLOEM et al., 2021). Dentre os principais distúrbios neurológicos, a DP é a que mais cresce em prevalência, incapacidade e mortes (GBD, 2017). De acordo com o *Global Burden of Disease Study* estima- se que em 2040 o número de indivíduos diagnosticados com a DP será de aproximadamente 13 milhões de pessoas no mundo (GBD, 2017; RAY DORSEY et al., 2018). Este número representa o dobro do número de casos registrados em 2015 GBD, 2017; RAY DORSEY et al., 2018), o que indica um rápido crescimento na incidência e duração da doença. Esse aumento vem sendo associado principalmente ao aumento da taxa de envelhecimento populacional (RAY DORSEY et al., 2018). Dessa forma, avanços na pesquisa clínica relacionada aos distúrbios da DP são imprescindíveis.

Estudos recentes sobre a patogênese da DP indicam que o início desta condição de saúde está associado ao depósito anormal de agregados proteicos ricos em α-sinucleína, denominados corpos de Lewy, no sistema nervoso central (BORGHAMMER, 2018; CÓPPOLA, 2018; RAZA et al., 2019). De acordo com o esquema de estadiamento proposto por Braak e colaboradores (2003), os primeiros locais de estadiamento da DP são comumente o bulbo olfatório, núcleo motor dorsal do vago e a região inferior do tronco cerebral (BORGHAMMER, 2018; BRAAK et al., 2003). Posteriormente são afetados os núcleos pontinhos monoaminérgicos e os neurônios dopaminérgicos da substancia negra (BORGHAMMER, 2018; BRAAK et al., 2003). Esse esquema de estadiamento explica a evolução clínica da doença de Parkinson, caracterizada pelo aparecimento inicial de sintomas não motores que incluem alterações como distúrbios do sono, hispomia e constipação intestinal (SCHAPIRA et al., 2017).

Os principais sintomas motores da DP são conhecidos como sinais cardinais e se referem ao tremor em repouso, bradicinesia, rigidez e instabilidade postural (AASETH et al., 2018; SCHAPIRA et al., 2017). Esses sintomas são predominantemente causados pela perda dos neurônios dopaminérgicos da substancia negra compacta no sistema nervoso central (SCHAPIRA et al., 2017). Por isso, uma das estratégias terapêuticas farmacológicas mais comumente utilizadas no tratamento da DP é a reposição de dopamina por meio do fármaco de administração oral Levodopa(POEWE et al., 2020; SCHAPIRA et al., 2017). De acordo com os critérios diagnósticos adotados pela *The United Kingdom Parkinson's Disease Society*, o diagnóstico da DP deve ser feito em três

etapas: 1) Diagnóstico de síndrome Parkinsoniana: presença de bradcinesia associada a outro sintoma dentre os sinais cardinais. 2) Exclusão de outros diagnósticos que podem causar a síndrome Parkinsoniana, como o Acidente Vascular Cerebral ou o Traumatismo Cranioencefálico e 3) Presença de três ou mais critérios de suporte como o início unilateral, tremor de repouso, progressão dos sintomas e melhora dos sintomas com o uso da Levodopa (MARSILI et al., 2018). Com a progressão da doença, comumente as doses de Levodopa também são aumentadas, resultando em períodos denominados "período *on*", no qual os sintomas motores estão atenuados, e "período *off*", no qual os sintomas motores reaparecem (SCHAPIRA et al., 2017). Entretanto, mesmo com as medidas farmacológicas de tratamento, o curso natural da doença resulta no declínio progressivo de diversas funções dos pacientes com DP, dentre elas a mobilidade (BOUCĄ-MACHADO et al., 2018; TOMLINSON et al., 2014). Nesses indivíduos, o declínio da mobilidade compromete a capacidade de locomoção de maneira segura e em diferentes ambientes e, comumente, passa a ser perceptível com a redução da velocidade de marcha, geralmente o primeiro sinal de alterações da marcha observadas nessa população(BOUCA-MACHADO et al., 2018).

Indivíduos com DP comumente apresentam redução da velocidade de marcha, diminuição do comprimento do passo, assimetria entre os membros, diminuição do balanço recíproco de membros superiores, aumento da base de suporte e da cadência e redução no equilíbrio postural durante a marcha (DI BIASE et al., 2020; MIRELMAN et al., 2019). Além disso, os indivíduos com DP podem apresentar hesitação para iniciar a marcha e episódios de congelamento ou "freezing", caracterizado por uma parada súbita transitória da atividade motora voluntária (DI BIASE et al., 2020; MIRELMAN et al., 2019). Com a progressão da doença, os padrões alterados de movimento na marcha também podem progredir, afetando a mobilidade, independência e a qualidade de vida destes indivíduos (DI BIASE et al., 2020; MIRELMAN et al., 2019).

Uma estratégia comumente utilizada na tentativa de melhorar a mobilidade de indivíduos com DP durante a marcha é a prescrição de dispositivos de auxílio, como bengalas e andadores (BRYANT et al., 2014). O uso desses dispositivos aumenta a base de apoio dos indivíduos, permitindo uma maior amplitude para o deslocamento do centro de massa durante a marcha (BRYANT et al., 2014). Assim, acredita-se que o uso desses dispositivos é capaz de melhorar o equilíbrio, a confiança e reduzir as quedas durante a marcha (BRYANT et al., 2014; KADER et al., 2018). Além disso, a utilização desses dispositivos assim como a percepção de necessidade do uso deles tende a aumentar entre

os indivíduos com DP ao longo dos anos (KADER et al., 2018). Entretanto, a prescrição do uso desses dispositivos é baseada apenas no julgamento clínico dos profissionais de saúde e nenhuma diretriz científica sobre as recomendações para o uso de dispositivos de auxílio para indivíduos com DP foi encontrada (BRYANT et al., 2014).

Em um estudo realizado com 85 indivíduos com DP, Bryant e colaboradores (2014) identificaram que 36,6% dos indivíduos utilizavam dispositivos de auxílio durante a marcha, sendo a bengala o dispositivo mais comum (BRYANT et al., 2014). Além disso, foi identificado que 56,8% dos indivíduos que demoraram mais de 13 segundos para realizar o teste *Timed Up Go* de cinco metros costumava utilizar dispositivos de auxílio. Entre os indivíduos que costumavam utilizar o dispositivo, 77,8% apresentaram menores níveis de confiança no equilíbrio (BRYANT et al., 2014). Entretanto, os autores não compararam o desempenho dos indivíduos nas atividades de marcha com e sem bengala (BRYANT et al., 2014). Além disso, o estudo não considerou qual era o tempo médio de utilização do dispositivo pelos indivíduos que relataram fazer uso dele (BRYANT et al., 2014).

Outros estudos se dedicaram a investigar os efeitos da utilização de dispositivos de auxílio durante a marcha em pacientes com DP (KEGELMEYER et al., 2013). Kegelmeyer e colaboradores (2013) investigaram a influência do uso de diferentes dispositivos de auxílio nos padrões de marcha em 27 indivíduos com DP (KEGELMEYER et al., 2013). De acordo com os autores somente a utilização do andador de quatro rodas reproduziu padrões de marcha semelhantes a marcha sem o dispositivo (KEGELMEYER et al., 2013). Para os outros dispositivos avaliados, (bengala de ponteira única, andador padrão e andador de duas e seis rodas) a utilização diminuiu a velocidade de marcha para todos os indivíduos (KEGELMEYER et al., 2013). Além disso, o uso dos dispositivos foi associado a diminuição do comprimento do passo, diminuição da base de suporte, aumento do tempo de duplo apoio e diminuição da fase de balanço durante a marcha (KEGELMEYER et al., 2013).

Em outro estudo, realizado por Bryant e colaboradores (2012), 10 indivíduos com DP foram avaliados durante a marcha sem dispositivo de auxílio, com bengala de ponteira única e com andador (BRYANT et al., 2012). Os autores reportaram que o uso dos dispositivos diminuiu a velocidade de marcha (BRYANT et al., 2012). Além disso, o uso do andador diminuiu o comprimento do passo durante a marcha. Já no estudo conduzido por Cubo e colaboradores (2003), 19 indivíduos com DP foram avaliados nas atividades de marcha sem uso de dispositivos de auxílio, marcha com andador padrão e marcha com

andador com rodas (CUBO et al., 2003). Os autores compararam o tempo de caminhada, o número e tempo de episódios de congelamentos e a duração média dos congelamentos (CUBO et al., 2003).Os autores identificaram que a velocidade de marcha foi maior sem a utilização dos dispositivos (CUBO et al., 2003). Além disso, a utilização do andador padrão aumentou significativamente o número e a duração do congelamento (CUBO et al., 2003).

Apesar dos resultados dos estudos supracitados indicarem que a utilização de dispositivos de auxílio à marcha pode piorar os padrões de marcha em indivíduos com DP, todos os estudos investigaram os efeitos imediatos do uso de tais dispositivos uma vez que os indivíduos receberam orientações e foram familiarizados com os dispositivos somente alguns minutos antes das medidas de avaliação(BRYANT et al., 2012; CUBO et al., 2003; KEGELMEYER et al., 2013). Dessa forma, apesar de serem comumente prescritos na prática clínica, os efeitos do treinamento e uso de dispositivos de auxílio, dentre eles a bengala de ponteira única, na velocidade de marcha de indivíduos com DP são pouco conhecidos.

OBJETIVOS

2.1 Objetivo primário

O objetivo primário deste projeto será investigar o efeito do treinamento e uso da bengala na velocidade de marcha de indivíduos com DP.

2.2 Objetivos secundários:

Os objetivos secundários serão: investigar o efeito do treinamento e uso da bengala na cadência, comprimento do passo, confiança na marcha, mobilidade, congelamento na marcha de indivíduos com DP e investigar a satisfação dos indivíduos com DP quanto ao uso da bengala.

MATERIAL E MÉTODO

Delineamento

Trata-se de um ensaio clínico aleatorizado com avaliadores cegados. O estudo será submetido ao Comitê de Ética em Pesquisa da Universidade Federal de Minas Gerais e o protocolo do estudo será registrado no www.ClinicalTrials.gov. O estudo seguirá as orientações *Consolidated Standards of Reporting Trials* (CONSORT) (CUSCHIERI, 2019).

Participantes

Indivíduos com diagnóstico de DP serão recrutados na comunidade na cidade de Belo Horizonte, Minas Gerais, Brasil. O recrutamento será feito por meio de listas de pacientes de outros projetos com a mesma população, além de divulgação nas mídias sociais, hospitais, unidades básicas de saúde e espaços da universidade. Serão incluídos indivíduos que atenderem aos seguintes critérios: idade ≥ 40 anos; diagnóstico de Parkinson confirmado por um neurologista; classificação de II a IV nos Estágios de Incapacidade de Hoehn & Yahr (HY)(GOULART et al., 2005); uso de medicação anti-parkinsoniana e que estejam medicamente estáveis por pelo menos 6 meses; capacidade de caminhar de maneira independente em um corredor de 14 metros com velocidade de marcha menor ou igual a 1,1 m/s e capacidade de utilizar uma bengala de ponteira única durante a marcha de maneira correta e segura sem ter feito a utilização prévia de dispositivos de auxílio. Para avaliar a capacidade do indivíduo de utilizar a bengala os seguintes critérios serão adotados: 1) ausência de comprometimento visual, cognitivo, físico ou auditivo que impossibilite o treinamento e uso do dispositivo e 2) ausência de perda do equilíbrio durante a marcha com o dispositivo (KEGELMEYER et al., 2013; MCCANDLESS et al., 2016). Indivíduos com alteração cognitiva, avaliada pelo Mini Exame do Estado Mental (BRUCKI et al., 2003), ou que apresentarem qualquer outra condição neurológica, cardiopulmonar ou musculoesquelética que possa comprometer a realização dos testes serão excluídos.

Randomização

Os indivíduos serão distribuídos aleatoriamente no grupo experimental e controle. A taxa de alocação será de 1:1, de acordo com o plano de aleatorização gerado por um website. A sequência de aleatorização será mantida em envelopes opacos selados. Os envelopes serão preparados antes do início do estudo por um assistente de pesquisa não envolvido no estudo.

Procedimentos

As avaliações dos indivíduos serão realizadas no NEUROLAB/UFMG. As avaliações serão conduzidas por um pesquisador treinado e cegado para a alocação dos grupos. Todos os indivíduos avaliados serão esclarecidos quanto aos objetivos do estudo e assinarão o Termo de Consentimento Livre e Esclarecido. Durante a avaliação inicial,

os critérios de elegibilidade serão verificados. Para os indivíduos que atenderem estes critérios, serão coletados os dados sociodemográficos (sexo, idade, escolaridade, nível socioeconômico e ocupação) e clínicos (tempo de evolução da doença, e *Unified Parkinson's Disease Rating Scale* (UPDRS))(GOULART et al., 2005). Para a escala UPDRS, será avaliada especificamente a sessão III, utilizada para avaliar o impacto da doença na função motora do indivíduo (SHEARIN et al., 2021; GOULART et al., 2005).

Os participantes incluídos serão aleatoriamente alocados para o grupo experimental ou grupo controle pelo fisioterapeuta responsável pela intervenção. Este fisioterapeuta estará cegado com relação à avaliação realizada e previamente treinado quanto aos procedimentos da intervenção. Após a alocação, os indivíduos do grupo intervenção serão orientados a manter seus cuidados à saúde habituais e receberão o treinamento de uso da bengala. O treinamento será realizado no NEUROLAB, UFMG, cuja anuência já foi obtida, e será ofertado em quatro sessões com duração de 40 minutos intercaladas em um período de 15 a 22 dias. Além disso, eles serão orientados a utilizar a bengala durante suas atividades cotidianas de locomoção, em ambiente interno e externo desde o primeiro dia do treinamento. Para acompanhar a adesão ao uso da bengala os indivíduos receberão um diário para registro do uso diário do dispositivo durante as atividades de locomoção.

Os indivíduos alocados no grupo controle serão orientados a manter os cuidados à saúde habituais e a não iniciar a utilização de qualquer dispositivo de auxílio a marcha no período de realização do estudo. Para controlar a quantidade de atenção recebida os indivíduos do grupo controle receberão uma intervenção placebo contendo alongamentos globais e educação em saúde. A intervenção será realizada no NEUROLAB, UFMGe será ofertada pelo mesmo fisioterapeuta responsável pelo treinamento do grupo experimental em quatro sessões com duração de 40 minutos intercaladas em um período de 15 a 22 dias.

Todos os indivíduos serão reavaliados após a randomização pelo mesmo fisioterapeuta que realizou a primeira avaliação, o qual estará cegado quanto ao grupo em que o indivíduo foi alocado. Os participantes serão orientados a não comentar com os avaliadores sobre o grupo para o qual foram alocados. Um mês após a reavaliação, os indivíduos serão novamente reavaliados (*follow-up*). Nestas duas reavaliações, serão coletados os dados das variáveis de desfechos principais. Os indivíduos do grupo experimental permanecerão com a bengala, mas nenhuma instrução adicional quanto ao uso será fornecida neste período entre a primeira reavaliação e a reavaliação de *follow-*

up. Todas as avaliações e treinamentos serão realizados no "período *on*" da medicação. Além disso, os dados vitais de todos os participantes serão monitorados antes e após cada sessão de intervenção e a presença de dor e/ou fadiga será questionada durante as intervenções.

Caso o grupo experimental apresente melhora do desfecho primário em relação ao grupo controle, será ofertada aos participantes do grupo controle a possibilidade de realizar o treinamento com a bengala após o término do estudo.

Intervenção do grupo experimental

A altura da bengala será ajustada para que o cotovelo de cada indivíduo fique a aproximadamente 30 graus de flexão (BRYANT et al., 2012). Quanto ao lado de utilização da bengala, os pacientes serão orientados a utilizar o dispositivo no lado de maior uso (dominante) ou com menor comprometimento (GLISOI et al., 2012).

O protocolo do treino de marcha será baseado nas Diretrizes de Exercício para Disfunção da Marcha em Doença de Parkinson (NI et al., 2018). Segundo essas Diretrizes, para desfechos relacionados a marcha, treinamentos específicos de marcha devem ser preferidos em relação programas gerais de exercício (NI et al., 2018). Além disso, treinamentos supervisionados são recomendados, pois estão associados a maior melhora da função motora (NI et al., 2018). Os autores ainda sugerem que o programa seja iniciado com velocidade de marcha confortável, auto selecionado, progredindo para velocidades máximas (NI et al., 2018). Assim, o treinamento será realizado com a supervisão de um fisioterapeuta e o protocolo de treinamento incluirá o treino de marcha em diferentes superfícies e velocidades, com a utilização da bengala, dividido nas seguintes etapas: 1) treino de marcha no solo em velocidade confortável, em superfície plana e estável (aquecimento); 2) treino de marcha no solo em velocidade confortável, em superfície íngreme e estável (rampa) 3) treino de marcha no solo em velocidade máxima, em superfície plana e estável 4) treino de marcha no solo em velocidade confortável, em superfície plana e estável (desaquecimento) As etapas 1 e 4 terão duração de 5 minutos cada. As etapas 3 e 4 terão duração de 10 minutos cada. Um intervalo será dado para o descanso do paciente entre as etapas e sempre que necessário. Além disso, no início de cada sessão de treinamento os indivíduos terão oportunidade de tirar possíveis dúvidas sobre o uso da bengala no seu contexto de vida diária.

Intervenção do grupo controle

O grupo controle receberá uma intervenção placebo baseada em protocolos de estudos prévios (Avelino et al., 2018; Martins et al.,2017). A intervenção incluirá alongamentos globais de membros superiores e membros inferiores e educação em saúde. Durante a intervenção será realizado 20 minutos de alongamentos globais estáticos e 20 minutos de orientações sobre cuidados com a saúde em geral. Os alongamentos serão realizados na frequência de três séries de 30 segundos e envolverão diferentes grupos musculares (Avelino et al., 2018; Martins et al.,2017). Na impossibilidade do paciente em realizar o auto alongamento os pesquisadores responsáveis pela implementação da intervenção fornecerão assistência. As orientações sobre cuidados com a saúde em geral incluirão informações sobre a DP e prevenção de quedas e serão baseadas na versão em português da Diretriz Europeia de Fisioterapia para a Doença de Parkinson (CAPATO et al., 2015).

Medidas de desfecho

Medidas de desfecho primária

<u>Velocidade de marcha</u>

A velocidade de marcha será medida pelo Teste de Velocidade de Marcha de 10 metros. Para isso, será utilizado um corredor com 14 metros onde os 10 metros centrais serão utilizados para calcular a velocidade de marcha. Os dois metros iniciais e os dois metros finais serão desconsiderados da avaliação pois correspondem ao tempo de aceleração e desaceleração do indivíduo durante a atividade de marcha. O indivíduo receberá a instrução de caminhar no espaço indicado primeiro em sua velocidade habitual e posteriormente em sua velocidade máxima. A velocidade de marcha será calculada através do tempo gasto para que o indivíduo percorra os 10m centrais. Este teste apresenta adequadas propriedades de medida para avaliação desse desfecho em indivíduos com DP e é amplamente utilizado na prática clínica (LIM et al., 2005). Um primeiro teste será realizado para a familiarização do paciente e logo em seguida um novo teste será feito para a registro da medida (Lindholm et al., 2018). Caso o indivíduo apresente algum episódio de congelamento durante o teste o número de episódios e o tempo de congelamento será registrado (Bloem, 2016).

Medidas de desfecho secundárias

Comprimento do passo e cadência:

Para mensuração do comprimento do passo e a cadência, o número de passos dados durante o teste de velocidade de 10m serão contabilizados (CAPATO et al., 2015). Para calcular a comprimento médio do passo, a distância total será dividida pelo número de passos. Para calcular a cadência o número de passos será dividido pelo tempo gasto para percorrer os 10 metros, em segundos (CAPATO et al., 2015).

Confiança na marcha

A confiança na marcha será avaliada pela *Gait Efficacy Scale-Brasil* (mGES-Brasil) (AVELINO et al., 2018). A mGES-Brasil é uma escala de dez itens que avalia a confiança do indivíduo durante a marcha em circunstâncias desafiadoras, como a marcha em diferentes superfícies (AVELINO et al., 2018). Cada item da escala é pontuado individualmente em uma escala Likert de zero a dez pontos, sendo pontuações maiores indicativos de maior confiança (AVELINO et al., 2018). A escala já foi adaptada transculturalmente para o português-Brasil e apresentou adequadas propriedades de medidas para avaliação deste desfecho (AVELINO et al., 2018).

Mobilidade funcional

A mobilidade funcional será avaliada pelo teste *Timed Up Go* (TUG). O TUG é um teste de fácil aplicação que mede o tempo gasto pelo indivíduo para se levantar de uma cadeira, caminhar 3 metros, virar e retornar para a cadeira (CAPATO et al., 2015). Este teste também apresenta adequadas propriedades de medida para avaliação desse desfecho em indivíduos com DP e um tempo de TUG > 8,5 segundos para realização do teste é associado ao maior risco de quedas em indivíduos com DP (CAPATO et al., 2015). Para mensuração deste desfecho um primeiro teste será realizado para a familiarização do paciente (CAPATO et al., 2015). Logo após o teste será realizado novamente e o tempo do segundo teste será registrado (CAPATO et al., 2015). Assim como para o teste de velocidade de marcha, o número e duração de possíveis episódios de congelamento serão registrados (Bloem, 2016).

Congelamento na marcha

O congelamento durante a marcha será avaliado pelo Questionário de Congelamento da Marcha (*Freezing of gait questionnaire*, FOG-Q). Esse questionário possui seis itens cujo objetivo é avaliar o congelamento durante a marcha em pacientes com DP (Baggio et al., 2012; Oliveira, 2010). O instrumento já foi traduzido transculturalmente para o português-Brasil (Baggio et al., 2012). Além disso, o questionário apresenta adequadas propriedades de medida e é considerado confiável para o rastreio e mensuração da gravidade do congelamento, assim como para avaliar este desfecho após intervenções (Baggio et al., 2012). Além do questionário, o número e duração de episódios de congelamento que os pacientes vierem a apresentar durante o teste de velocidade de marcha e o TUG será reportado.

Satisfação com o uso da bengala

A satisfação em relação ao uso da bengala será coletada nos indivíduos do grupo experimental ao final da participação no estudo. Para isso, será utilizado o *Quebec User Evaluation of Satisfaction with Assistive Technology (QUEST 2.0)* desenvolvido com o objetivo de avaliar a satisfação do usuário com a tecnologia assistiva em diversos aspectos como conforto, segurança, peso, facilidade de uso, etc (Carvalho et al., 2014). O questionário possui 12 itens, pontuados de zero a cinco onde maiores pontuações refletem maior satisfação do paciente com a tecnologia assistiva (Carvalho et al., 2014). Este instrumento já foi traduzido transculturalmente para o português-Brasil e se mostrou confiável e válido para a medição da satisfação de usuários de tecnologia assistiva de auxílio a marcha (Carvalho et al., 2014).

Cálculo amostral

Vinte e seis indivíduos serão incluídos no presente estudo. O cálculo do tamanho da amostra foi realizado considerando a diferença clinicamente importante da medida de desfecho primária velocidade de marcha (Hass et al., 2014). Considerando um nível de significância (α) de 5% e o power de 0,80, foi encontrado um n=9 para cada grupo. Portanto, um tamanho amostral de 18 indivíduos. Assumindo-se uma perda amostral de 30% dos indivíduos ao longo do desenvolvimento do estudo determinou-se uma amostra de 26 indivíduos no total, sendo 13 em cada grupo.

Análise dos dados

A análise será realizada por um pesquisador independente, cegado em relação a aleatorização dos grupos, utilizando a análise de intenção de tratar e por protocolo. A normalidade dos dados será testada para todas as variáveis numéricas contínuas. Para caracterização da amostra e para a investigação da satisfação com o uso da bengala serão utilizadas estatísticas descritivas. A diferença entre os grupos para as demais variáveis desfecho (velocidade de marcha, confiança na marcha, cadência, comprimento do passo, mobilidade funcional, congelamento da marcha) será avaliada utilizando ANOVA de medidas repetidas, considerando as medidas coletadas na avaliação inicial, após o treinamento e no *follow-up*. Todas as análises estatísticas serão realizadas utilizando o software estatístico SPSS (SPSS Inc., Chicago, IL, Estados Unidos). Para todas as análises inferenciais será estabelecido um nível de significância de α=5%.

Referências

Aaseth J, Dusek P, Roos PM. Prevention of progression in Parkinson's disease. Biometals. 2018;31(5):737-747. doi:10.1007/s10534-018-0131-5

Avelino PR, Menezes KKP, Nascimento LR, et al. Cross-cultural adaptation of the Modified Gait Efficacy Scale for individuals with stroke. Rev Ter Ocup Univ Sao Paulo. 2018 ;29(3):230-6. https://doi.org/10.11606/issn.2238-6149.v29i3p230-236

Avelino PR, Nascimento LR, Menezes KKP, Scianni AA, Ada L, Teixeira-Salmela LF. Effect of the provision of a cane on walking and social participation in individuals with stroke: protocol for a randomized trial. Braz J Phys Ther. 2018;22(2):168-173. doi: 10.1016/j.bjpt.2017.11.002.

Baggio JA0, Curtarelli MB, Rodrigues GR, et al. Validity of the Brazilian Version of the Freezing of Gait Questionnaire. Arquivos de Neuro-Psiquiatria. 2012 (70)8. doi.org/10.1590/S0004-282X2012000800008>.

Bloem BR, Marinus J, Almeida Q, Dibble L, Nieuwboer A, Post B, Ruzicka E, Goetz C, Stebbins G, Martinez-Martin P, Schrag A; Movement Disorders Society Rating Scales Committee. Measurement instruments to assess posture, gait, and balance in Parkinson's disease: Critique and recommendations. Mov Disord. 2016 Sep;31(9):1342-55. doi: 10.1002/mds.26572.

Bloem BR, Okun MS, Klein C. Parkinson's disease. Lancet. 2021;397(10291):2284-2303. doi:10.1016/S0140-6736(21)00218-X

Borghammer P. How does parkinson's disease begin? Perspectives on neuroanatomical pathways, prions, and histology. Mov Disord. 2018;33(1):48-57.doi:10.1002/mds.27138

Bouça-Machado R, Maetzler W, Ferreira JJ. What is Functional Mobility Applied to Parkinson's Disease?. J Parkinsons Dis. 2018;8(1):121-130. doi:10.3233/JPD-171233

Braak H, Del Tredici K, Rüb U, et al. Staging of brain pathology related to sporadic Parkinson's disease. Neurobiol Aging. 2003;24(2):197-211. doi:10.1016/s0197-4580(02)00065-9

Brucki SM, Nitrini R, Caramelli P, et al. Sugestões para o uso do mini-exame do estado mental no Brasil. Arq Neuropsiquiatr. 2003;61(3B):777-781. doi:10.1590/s0004-282x2003000500014

Bryant MS, Pourmoghaddam A, Thrasher A. Gait changes with walking devices in persons with Parkinson's disease. Disabil Rehabil Assist Technol. 2012;7(2):149-152. doi:10.3109/17483107.2011.602461

Bryant MS, Rintala DH, Graham JE, Hou JG, Protas EJ. Determinants of use of a walking device in persons with Parkinson's disease. Arch Phys Med Rehabil. 2014;95(10):1940-1945. doi:10.1016/j.apmr.2014.06.002

Capato TTC, Domingos JMM, Almeida LRS. Versão Em Português Da Diretriz Europeia de Fisioterapia Para a Doença de Parkinson. Omnifarma; 2015. Available from: https://www.parkinsonnet.nl/app/uploads/sites/3/2019/11/diretriz_dp_brasil_versao_final_publicada.pdf

Carvalho KE, Gois Júnior MB, Sá KN. Tradução e validação do Quebec User Evaluation of Satisfaction with Assistive Technology (QUEST 2.0) para o idioma português do Brasil. Rev Bras Reumatol. 2014;54(4):260-267. doi:10.1016/j.rbr.2014.04.003

Cóppola VS. Agregação de alfa-Sinucleína na Doença de Parkinson: importância do estresse de retículo endoplasmático. Tese (doutorado) - Universidade Federal do Paraná, Setor de Ciências Biológicas, Programa de Pós-Graduação em Biologia Celular e Molecular. Defesa: Curitiba, 2018. Disponível em: https://acervodigital.ufpr.br/handle/1884/70618?show=full

Cubo E, Moore CG, Leurgans S, Goetz CG. Wheeled and standard walkers in Parkinson's disease patients with gait freezing. Parkinsonism Relat Disord. 2003;10(1):9-14. doi:10.1016/s1353-8020(03)00060-9

Cuschieri S. The CONSORT statement. Saudi J Anaesth. 2019;13(Suppl 1):S27-S30. doi:10.4103/sja.SJA_559_18

di Biase L, Di Santo A, Caminiti ML, et al. Gait Analysis in Parkinson's Disease: An Overview of the Most Accurate Markers for Diagnosis and Symptoms Monitoring. Sensors (Basel). 2020;20(12):3529. Published 2020 Jun 22. doi:10.3390/s20123529

GBD 2015 Neurological Disorders Collaborator Group. Global, regional, and national burden of neurological disorders during 1990-2015: a systematic analysis for the Global Burden of Disease Study 2015. Lancet Neurol. 2017;16(11):877-897. doi:10.1016/S1474-4422(17)30299-5

Glisoi SFN, Ansai JH, Silva TO, et al. Auxiliary devices for walking: guidance, demands and falls prevention in elderly. Geriatr Gerontol Aging. 2012 6(3):261-72. Disponível em: https://cdn.publisher.gn1.link/ggaging.com/pdf/v6n3a06.pdf

Goulart F, Pereira LX, Goulart DF. Uso de escalas para avaliação da Doença de Parkinson em Fisioterapia. Fisioterapia e Pesquisa. 2005; 11(1) doi.org/10.1590/fpusp.v11i1.76385

Hass CJ, Bishop M, Moscovich M, et al. Defining the clinically meaningful difference in gait speed in persons with Parkinson disease. Journal of Neurologic Physical Therapy. 2014;38(4):233-238. doi:10.1097/NPT.0000000000000055

Kader M, Jonasson SB, Iwarsson S, et al. Mobility device use in people with Parkinson’s disease: A 3-year follow-up study. Acta Neurologica Scandinavica. 2018;138(1):70-77. doi:10.1111/ane.12942

Lim LIIK, van Wegen EEH, de Goede CJT, et al. Measuring gait and gait-related activities in Parkinson’s patients own home environment: a reliability, responsiveness and

feasibility study. Parkinsonism & Related Disorders. 2005;11(1). doi:10.1016/j.parkreldis.2004.06.003

Martins JC, Aguiar LT, Nadeau S, Scianni AA, Teixeira-Salmela LF, Faria CDCM. Efficacy of Task-Specific Training on Physical Activity Levels of People With Stroke: Protocol for a Randomized Controlled Trial. Phys Ther. 2017 Jun 1;97(6):640-648. doi: 10.1093/physth/pzx032.

Marsili L, Rizzo G, Colosimo C. Diagnostic criteria for Parkinson's disease: From James Parkinson to the concept of prodromal disease. Frontiers in Neurology. 2018;9(MAR). doi:10.3389/fneur.2018.00156

McCandless PJ, Evans BJ, Janssen J, Selfe J, Churchill A, Richards J. Effect of three cueing devices for people with Parkinson's disease with gait initiation difficulties. Gait Posture. 2016;44:7-11. doi:10.1016/j.gaitpost.2015.11.006

Ni M, Hazzard JB, Signorile JF, Luca C. Exercise Guidelines for Gait Function in Parkinson's Disease: A Systematic Review and Meta-analysis. Neurorehabilitation and Neural Repair. 2018;32(10):872-886. doi:10.1177/1545968318801558

Oiveira JA. Validação da versão brasileira da escala de equilíbrio e marcha (GABS) e análise do risco de quedas em indivíduos com Doença de Parkinson e sujeitos saudáveis. 2010. 10.11606/D.17.2010.tde-18122014-101553

Poewe W, Espay AJ. Long duration response in Parkinson's disease: Levodopa revisited. Brain. 2020;143(8):2332-2335. doi:10.1093/brain/awaa226

Ray Dorsey E, Elbaz A, Nichols E, et al. Global, regional, and national burden of Parkinson's disease, 1990–2016: a systematic analysis for the Global Burden of Disease Study 2016. The Lancet Neurology. 2018;17(11):939-953. doi:10.1016/S1474-4422(18)30295-3

Raza C, Anjum R, Shakeel N ul A. Parkinson's disease: Mechanisms, translational models and management strategies. Life Sciences. 2019;226:77-90. doi:10.1016/j.lfs.2019.03.057

Schapira AHV, Chaudhuri KR, Jenner P. Non-motor features of Parkinson disease. Nature Reviews Neuroscience. 2017;18(7):435-450. doi:10.1038/nrn.2017.62

Shearin S, Medley A, Trudelle-Jackson E, Swank C, Querry R. Differences in predictors for gait speed and gait endurance in Parkinson's disease. Gait Posture. 2021;87:49-53. doi:10.1016/j.gaitpost.2021.04.019

Tomlinson CL, Herd CP, Clarke CE, et al. Physiotherapy for parkinson's disease: A comparison of techniques. Cochrane Database of Systematic Reviews. 2014;2014(6):1-119. doi:10.1002/14651858.CD002815.pub2

ORÇAMENTO FINANCEIRO

Materiais	Custo unitário	Custo total
04 pacotes de folha de Ofício tamanho A4	R$26,99	R$107,96
04 cartuchos para impressora	R$90,00	R$360,00
13 Bengalas de ponteira única	R$50,00	R$650,00
Total: R$ 1117,96		

Todos os gastos deste estudo serão assumidos pelos pesquisadores responsáveis.

CRONOGRAMA

Período	2023/2	2024/1	2024/2	2025/1	2025/2	2026/1	2026/2
Atualização bibliográfica	■	■	■	■	■	■	■
Cumprimento dos créditos	■	■	■	■			
Submissão do projeto ao Comitê de Ética	■	■	■				
Submissão do projeto as agências de fomento	■	■	■				
Submissão do protocolo para publicação			■	■			
Período de recrutamento				■	■	■	
Avaliação inicial				■	■	■	
Reavaliação				■	■	■	
Follow-up				■	■	■	
Análise estatística parcial e final e interpretação dos resultados					■	■	
Preparação e defesa da tese						■	■
Encaminhamento para publicação							■

Observação: o recrutamento e a coleta dos dados serão iniciados apenas após a aprovação do COEP. Portanto, pode haver ajustes neste cronograma a depender da data de aprovação do COEP**.**

APÊNDICE 1- TERMO DE CONSENTIMENTO LIVRE E ESCLARECIDO

TERMO DE CONSENTIMENTO LIVRE E ESCLARECIDO

TERMO DE CONSENTIMENTO LIVRE E ESCLARECIDO Nº________

TÍTULO DO PROJETO DE PESQUISA: EFEITO DO TREINAMENTO E USO DA BENGALA NA MOBILIDADE DE INDIVÍDUOS COM DOENÇA DE PARKINSON: UM ENSAIO CLÍNICO ALEATORIZADO

INVESTIGADORAS:
- Profa. Christina Danielli Coelho de Morais Faria, fisioterapeuta, Ph.D. Professora do Departamento de Fisioterapia da Universidade Federal de Minas Gerais (UFMG). Telefone: (31) 3409-7448; (31) 3409-4783; cdcmf@ufmg.br
- Jordana de Paula Magalhães, fisioterapeuta, aluna do Programa de Pós- Graduação em Ciências da Reabilitação da UFMG. Telefone: (31) 987733602; jordanamagalhaes.jpm@gmail.com

INFORMAÇÕES:
Você está sendo convidado a participar de um projeto de pesquisa intitulado: “Efeito do treinamento e uso da bengala na mobilidade de indivíduos com Doença de Parkinson: um ensaio clínico aleatorizado” a ser desenvolvido pelo Departamento de Fisioterapia da Escola de Educação Física,Fisioterapia e Terapia Ocupacional da Universidade Federal de Minas Gerais. Embora sejam comumente utilizadas por indivíduos com Doença de Parkinson, o efeito do treinamento e uso da bengala na mobilidade desses pacientes não é conhecido. Portanto, o objetivo deste projeto de pesquisa é investigar o efeito do treinamento e uso da bengala na velocidade de marcha, confiança na marcha, cadência, comprimento do passo, mobilidade e congelamento na marcha de indivíduos com Doença de Parkinson e investigar a satisfação dos indivíduos com o uso do dispositivo.

DESCRIÇÃO DOS TESTES E DAS INTERVENÇÕES A SEREM REALIZADAS

Avaliação inicial
Caso você concorde em participar, inicialmente será realizada uma entrevista para a coleta dos seus dados pessoais seguida de um exame físico, ambos administrados por um examinador previamente treinado . Para isso, será utilizado um questionário previamente estruturado que incluirá algumas perguntas sobre você e sobre a sua condição de saúde. Além disso, você realizará alguns testes comumente utilizados na prática clínica para obter informações sobre a sua velocidade de marcha, confiança na marcha, cadência, comprimento do passo, mobilidade e congelamento durante a marcha. Durante todos os procedimentos, serão consideradas a sua segurança, bem estar e conforto. Os avaliadores respeitarão as medidas de prevenção de disseminação doenças infectocontagiosas como o uso de máscara cirúrgica descartável e disponibilização de álcool em gel 70% para higienização das mãos.

Grupos do estudo

Ao entrar nesse projeto de pesquisa você será sorteado para participar de um dos seguintes grupos: 1) grupo intervenção: Os indivíduos do grupo intervenção receberão uma bengala de ponteira única que deverá ser utilizada durante todas as suas atividades de locomoção durante 22 dias. Os indivíduos receberão quatro sessões de treinamento de uso da bengala neste período. Durante as sessões de treinamento os indivíduos poderão tirar qualquer dúvida sobre o uso do dispositivo bem como treinar o seu uso em diferentes atividades de marcha. As sessões de treinamento terão duração de 40 minutos e serão administradas por um fisioterapeuta treinado. Além disso os indivíduos receberão um diário para registrar diariamente o uso da bengala nas atividades de locomoção 2) grupo controle: A intervenção do grupo controle consiste em alongamentos globais e educação em saúde. A intervenção será ofertada em quatro sessões com duração de 40 minutos intercaladas em um período de 15 a 22 dias e serão administradas por um fisioterapeuta treinado. Ambos os grupos deverão manter os seus cuidados habituais de saúde. Caso o grupo experimental apresente melhora ao final do estudo, será ofertada aos participantes do grupo controle a possibilidade de realizar o treinamento com a bengala realizado pelo grupo experimental.

Procedimentos

Inicialmente, será realizada uma avaliação inicial onde algumas medidas serão avaliadas e você responderá alguns questionários e desempenhará testes de avaliação da marcha e mobilidade. Em seguida, você será alocado em um dos grupos e receberá as orientações pertinentes ao grupo de alocação. Os mesmos procedimentos da avaliação inicial, ou seja, todos os testes e medidas empregadas, serão realizados novamente após 22 dias da primeira avaliação e no acompanhamento de um mês após a segunda avaliação. Todos os procedimentos, testes, medidas e intervenções a serem realizados no presente estudo são padronizados e comumente adotados na prática clínica ou em estudos científicos já realizados anteriormente. Durante todos os procedimentos, serão considerados a sua segurança, bem estar e conforto.

Riscos

Os ricos da participação neste estudo incluem dores musculares durante e após a execução dos testes, pois estes podem exigir um esforço físico maior do que aquele que os indivíduos realizam diariamente.Caso isso ocorra, os participantes serão assistidos pelo pesquisador responsável pelo tempo necessário utilizando procedimentos recomendados. Além disso, os participantes poderão sentir-se cansados e fadigados durante a realização das avaliações ou do treinamento. Para minimizar a ocorrência deste desconforto, será realizado um períodode descanso entre os testes e durante o treinamento. Qualquer tipo de desconforto vivenciado pelo participante deve ser informado para que os pesquisadores tomem as devidasprovidências com o objetivo de minimizá-lo.

Benefícios

Você e futuros pacientes poderão se beneficiar com os resultados desse estudo, principalmente porque o objetivo principal do mesmo é investigar os efeitos do uso de um dispositivo comumente utilizado por indivíduos com Doença de Parkinson.

A partir das informações obtidas neste estudo, será possível conhecer os efeitos do uso da bengala na velocidade de marcha, confiança na marcha, cadencia e comprimento do passo, mobilidade e congelamento na marcha desses indivíduos. Além disso, esse estudo também irá obter informações sobre a satisfação com o uso da bengala pelos pacientes do grupo experimental.

Confidencialidade

Para garantir o seu anonimato e a confidencialidade dos seus dados este estudo utilizará uma senha numérica para sua identificação. Esta senha será utilizada em todos os seus testes, não sendo utilizado o seu nome. Caso informações originadas deste estudo forem publicadas em revista ou evento científico, você não será reconhecido individualmente, pois será representado pela senha. Os dados coletados neste estudo serão armazenados durante um período de dez anos.

Natureza voluntária do estudo e pagamento
Sua participação neste estudo é inteiramente voluntária e você é livre para concordar ou não com a participação. Caso desejado, você poderá abandonar o estudo a qualquer momento, sem que isto lhe traga qualquer prejuízo pessoal. A participação no estudo não acarretará custos para você e não haverá nenhuma forma de pagamento pela sua participação no estudo. Os testes, e todos os materiais utilizados na pesquisa, incluindo a bengala de ponteira única, não terão custo para você. Caso seja necessário, gasto adicionais com transporte serão de responsabilidade dos pesquisadores.
Depois de ter lido as informações acima, se for de sua vontade participar, por favor, preencha e assine esse documento, em duas vias, nos espaços reservados e em todas as páginas que compõe esse documento. Uma das vias desse documento ficarána posse do participante e a outra via na posse do pesquisador.

DECLARAÇÃO E ASSINATURA
Eu,____li e entendi toda a informação repassada sobre o estudo, sendo que os objetivos, procedimentose linguagem técnica foram satisfatoriamente explicados. Tive tempo suficiente para considerar as informações acima e tive a oportunidade de tirar todas as minhas dúvidas. Estou assinando duas vias deste termo voluntariamente, sendo uma via do documento para mim e outra para os pesquisadores, e tenho direito de, agora ou mais tarde, discutir qualquer dúvida que venha a ter com relação à pesquisa com:

- Christina Danielli Coelho de Morais Faria, fisioterapeuta, Ph.D. Professora do Departamento de Fisioterapia da Universidade Federal de Minas Gerais (UFMG). Telefone: (31) 3409-7448; (31) 3409-4783; cdcmf@ufmg.br
- Jordana de Paula Magalhães, fisioterapeuta, aluna do Programa de Pós- Graduação em Ciências da Reabilitação da UFMG. Telefone: (31) 987733602; jordanamagalhaes.jpm@gmail.com

Além disso, em caso de dúvidas éticas você tem direito de entrar em contato com: Comitê de Ética em Pesquisa da UFMG: (31) 3409-4592. Endereço: Av. Antônio Carlos, 6627 Unidade Administrativa II, sala 2005. Campus Pampulha, BH/MG. CEP 31270-901.

Assinando esse termo de consentimento, estou indicando que concordo em participardeste estudo.

____ ________________________

Assinatura do Participante Data
RG:____________________________CPF:____
End.:____
____

____ ______________________

Assinatura do Investigador Responsável Data
Christina Danielli Coelho de Morais Faria
Jordana de Paula Magalhães

APÊNDICE II -FICHA DE AVALIAÇÃO

PROJETO DE PESQUISA:

EFEITO DO TREINAMENTO E USO DA BENGALA NA MOBILIDADE DE INDIVÍDUOS COM DOENÇA DE PARKINSON: UM ENSAIO CLÍNICO ALEATORIZADO

FICHA DE AVALIAÇÃO (incicial, reavaliação ou follow-up): ________________________

DATA:___________________ HORÁRIO:________________CÓDIGO:___________________

1. DADOS DEMOGRÁFICOS

1.1 Nome:____
1.2 Endereço: ____
1.2 Telefone:____ Sexo: ( ) Feminino ( ) Masculino
1.3 Data de Nascimento:______________________________ Idade:________________________
1.4 Estado civil:_____________________ Mora com:_____________________________________
1.5 Escolaridade (anos estudados):___________________ Formação:________________________
1.6 Ocupação: ____
1.7 Acompanhante (nome e contato):____

2 DADOS CLÍNICOS DA DOENÇA DE PARKINSON

2.1 Tempo desde início dos sintomas:___________Tempo de diagnóstico:_______________
2.2 Diagnóstico por Neurologista: ( ) SIM ( ) NÃO
2.3 Lado mais comprometido:_____________Uso de estimulação profunda: ( ) SIM ( ) NÃO
2.4 HY:______________UPDRS-III: ____________

3 DADOS CLÍNICOS GERAIS

3.1 MS dominante:_____________MI dominante: _______________
3.2 Déficit visual: ( ) Não ( ) Sim Déficit auditivo: ( ) Não ( ) Sim
3.3 Deambula de forma independente: ( ) Não ( ) Sim
3.4 Número de doenças associadas (descrição):____
____

3.5 Quedas: Quantas vezes você caiu na última semana? Mês? Nos últimos 6 meses?
____

3.6 Você está realizando algum exercício físico atualmente? ( ) Não ( ) Sim Qual? _________________
Quantas vezes na semana? ________________. Qual a duração (minutos)?______________________
3.7 Você está realizando algum tratamento de reabilitação? (ex:fisioterapia, terapia ocupacional, fonoaudiologia, etc) ( ) Não ( ) Sim Qual? ______________________
Quantas vezes na semana? ________________ Qual a duração (minutos): ____________________

3.8 Medicamentos em uso

Medicamento	Dose	Horário	Tempo de uso

Peso (Kg): ____________ Altura (cm): _______________
PA: _______________ FC: ________________ FR: ______________ SatO2: _________________

Capaz de utilizar bengala de ponteira única: ( ) Sim ( ) Não Motivo: ________________________

MINI EXAME DO ESTADO MENTAL

Orientação temporal		Pontos	Pontuação
**Que dia é hoje?**		1	
**Em que mês estamos?**		1	
**Em que ano estamos?**		1	
**Em que dia da semana estamos?**		1	
**Qual a hora aproximada?**	Considere a variação de uma ou menos 1 hora	1	
**Orientação espacial**		Pontos	Pontuação
**Em que local nós estamos?**	Consultório, dormitório, sala - apontando para o chão	1	
**Que local é este aqui?**	Apontando ao redor num sentido mais amplo: hospital, casa de repouso, própria casa	1	
**Em que bairro nós estamos ou qual o nome de uma rua próxima.**		1	
**Em que cidade nós estamos?**		1	
**Em que Estado nós estamos?**		1	
**Memória imediata**		Pontos	Pontuação
**Eu vou dizer três palavras e você irá repeti-las a seguir: carro, vaso, tijolo**	Dê 1 ponto para cada palavra repetida acertadamente na 1ª vez, embora possa repeti-las até três vezes para o aprendizado, se houver erros.	3	
**CALCULO**		Pontos	Pontuação
Subtração de setes seriadamente: **Quanto é: 100-7, 93-7, 86-7, 79-7, 72-7, 65**	Considere 1 ponto para cada resultado correto. Se houver erro, corrija-o e prossiga. Considere correto se o examinado espontaneamente se autocorrigir. **(VER*)**	5	
**Evocação das palavras**		Pontos	Pontuação
**Quais as palavras que você acabou de repetir?**	*Pergunte quais as palavras que o sujeito acabara de repetir - 1 ponto para cada*	3	
**Nomeação**		Pontos	Pontuação
**Que objeto é este?**	Peça para o sujeito nomear os objetos mostrados (relógio, caneta) - 1 ponto para cada.	2	
**Repetição**		Pontos	Pontuação
**Preste atenção: vou lhe dizer uma frase e quero que você repita depois de mim: "Nem aqui, nem ali nem lá".**	Considere somente se a repetição for perfeita (1 ponto)	1	
**Comando**		Pontos	Pontuação
**"Pegue este papel com sua mão direita (1 ponto), dobre-o ao meio (1 ponto) e coloque-o no chão (1 ponto)".**	Total de 3 pontos. Se o sujeito pedir ajuda no meio da tarefa não dê dicas.	3	
**Leitura**	Mostre a frase escrita 'FECHE OS OLHOS' e peça para o indivíduofazer o que está sendo mandado. Não auxilie se pedir ajuda ou se só ler a frase sem realizar o comando.	1	
**Frase** Escreva uma frase	Peça ao indivíduo para escrever uma frase. Se não compreender osignificado, ajude com: alguma frase que tenha começo, meio e fim; alguma coisa que aconteceu hoje; alguma coisa que queira dizer. Paraa correção não são considerados erros gramaticais ou ortográficos	1	
**Cópia do desenho:** Faça uma cópia deste desenho o melhor possível	Mostre o modelo e peça para fazer o melhor possível. Considere apenas se houver 2 pentágonos interseccionados (10 ângulos) formando uma figura de quatro lados ou com dois ângulos (1 ponto)	1	
**TOTAL**	30		

*Soletrar a palavra MUNDO de trás para frente. *- um ponto para cada letra na posição correta - *Obs: Será considerado apenas a notareferente ao melhor desempenho*

PONTOS DE CORTE
13 para analfabetos
18 para escolaridade baixa/média (1a 7 anos de escolaridade)
26 para alta escolaridade (8 ou maisanos de escolaridade)

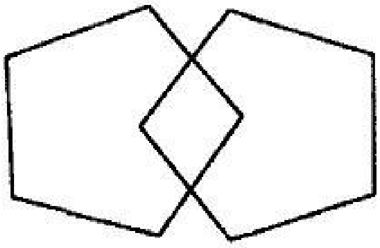

UNIFIED PARKINSON'S DISEASE RATE SCALE (UPDRS)

(PARCIAL)

III- EXPLORACÃO MOTORA

1. Linguagem falada
0= Normal.
1= Leve perda de expressão dicção e/ou volume da voz.
2= Monótona, arrastada, mas compreensível; alteração moderada.
3= Alteração marcada, difícil de entender.
4= Ininteligível

2. Expressão facial
0= Normal
1= Hiponímia mínima; poderia ser normal ("cara de jogador de pôquer")
2= Diminuição leve mas claramente anormal da expressão facial.
3= Hiponímia moderada; lábios separados em algumas ocasiões.
4= Face fixa ou em máscara com perda grave ou total da expressão facial, lábios separados ≥ 0,6cm)

3. Tremor em repouso
0= Ausente
1=Leve e pouco frequente
2= De pequena amplitude e continuo ou de amplitude moderada e aparição intermitente.
3= De amplitude moderada e presente quase continuamente.
4= De amplitude marcada e presente quase continuamente.

4. Tremor de ação ou postural das mãos:
0= Ausente
1=Leve; presente durante a atividade
2=De amplitude moderada, presente durante a atividade.
3=De amplitude moderada, presente ao manter uma postura assim como durante a atividade.
4=De amplitude marcada, dificulta a alimentação.

5. Rigidez: (Avaliada através da mobilização passiva das articulações maiores, com o paciente sentado e relaxado. Não avaliar o fenômeno da roda denteada).
0= Ausente
1=Leve só percebida quando ativada por movimentos contralaterais ou outros movimentos.
2= Leve a moderada.
3= Marcada, mas permite alcançar facilmente a máxima amplitude de movimento.
4= Grave, a máxima amplitude do movimento é alcançada com dificuldade.

6. Destreza digital. (O paciente bate o polegar contra o indicador rápida e sucessivamente com a maior amplitude possível; cada mão separadamente).
0= Normal
l= Ligeiramente lento e/ou redução da amplitude.
2= Alteração moderada. Fadiga clara e precoce. O movimento pode se deter ocasionalmente.
3= Alteração grave. Freqüente indecisão ao iniciar o movimento ou paradas enquanto realiza o movimento.
4= Apenas pode realizar o exercício.

7. Movimentos das mãos. (O paciente abre e fecha a mão rápida e sucessivamente com a maior amplitude possível; cada mão separadamente).

0= Normal
l= Lentidão leve e/ou redução da amplitude.
2= Alteração moderada. Fadiga clara e precoce. O movimento pode se deter ocasionalmente.
3= Alteração grave. Freqüente indecisão em iniciar o movimento ou paradas enquanto realiza o movimento.
4= Apenas pode realizar o exercício.

8. Movimentos das mãos rápidos e alternantes: (Movimentos de pronação-supinação, vertical ou horizontalmente com a maior amplitude possível e ambas as mãos simultaneamente).

0= Normal
l= Lentidão leve e/ou redução da amplitude
2= Alteração moderada. Fadiga clara e precoce. O movimento pode se deter ocasionalmente.
3= Alteração grave. Freqüente indecisão ao iniciar o movimento ou paradas enquanto realiza o movimento.
4= Apenas pode realizar o exercício.

9. Agilidade das pernas: (O paciente bate o calcanhar contra o solo em sucessão rápida, levantando a perna por completo. A amplitude deveria situar-se em 7 a 8 cm.)

0= Normal
1= Lentidão leve e/ou redução da amplitude.
2=Alteração moderada. Fadiga clara e precoce. O movimento pode se deter ocasionalmente.
3= Alteração grave. Freqüente indecisão ao iniciar o movimento ou paradas enquanto realiza o movimento.
4= Apenas pode realizar o exercício.

10. Levantar de uma cadeira. (O paciente tenta levantar-se de uma cadeira de madeira ou metal de encosto vertical mantendo os braços cruzados sobre o tórax)

0= Normal
1=Lento ou necessita de mais de uma tentativa.
2= Levanta-se com apoio nos braços da cadeira.
3= Tende a cair para trás e pode tentar várias vezes ainda que se levante sem ajuda.
4= Não pode se levantar da cadeira sem ajuda.

11. Postura

0= Erguido normalmente.
1 = Não totalmente erguido, levemente encurvado, pode ser normal em pessoas idosas.
2= Postura moderadamente encurvada, claramente anormal, pode estar inclinado ligeiramente para um lado.
3=Postura intensamente encurvada com cifose; pode estar inclinado moderadamente para um lado.
4=Flexão marcada com extrema alteração postural

12. Marcha

0= Normal
1= A marcha é lenta, pode arrastar os pés e os passos podem ser curtos, mas não existe propulsão nem festinação.
2= Caminha com dificuldade, mas necessita pouca ou nenhuma ajuda; pode existir certa festinação, passos curtos ou propulsão.
3=Grave transtorno da marcha que exige ajuda.
4=A marcha é impossível, ainda que com ajuda.

13. Estabilidade postural (Observa-se a resposta a um deslocamento súbito para trás, provocado por um empurrão nos ombros, estando o paciente em pé com os olhos abertos e os pés ligeiramente separados. Avisar o paciente previamente)
0= Normal
1=Retropulsão, ainda que se recupera sem ajuda.
2=Ausência de reflexo postural; poderia ter caído se o avaliador não impedisse.
3= Muito instável; tendência a perder o equilíbrio espontaneamente.
4= Incapaz de manter-se de pé sem ajuda.

14. Bradicinesia e hipocinesia. (Combinação de lentidão, indecisão, diminuição da oscilação dos braços, redução da amplitude dos movimentos e escassez de movimentos em geral).
0= Ausente
1= Lentidão mínima, dando ao movimento um caráter decidido; poderia se normal em algumas pessoas. Amplitude possivelmente reduzida.
2= Grau leve de lentidão e escassez de movimentos; evidentemente anormal. Pode haver diminuição da amplitude.
3= Lentidão moderada, pobreza de movimentos ou amplitude reduzida dos mesmos.
4= Lentidão marcada e pobreza de movimentos com amplitude reduzida dos mesmos

**- Escala de estágios de incapacidade de Hoehn e Yahr (modificada):**
0: Ausência de sinais da doença
1,0: Alteração unilateral
1,5: Alteração unilateral com comprometimento axial
2,0: Alteração bilateral, sem déficit de equilíbrio
2,5: Alteração bilateral leve com recuperação na prova do empurrão
3,0: Alteração bilateral leve a moderada, certa instabilidade postural,fisicamente independente.
4,0: Incapacidade grave, ainda capaz de caminhar ou permanecer de pésem ajuda.
5,0: Confinado à cama ou cadeira de rodas a não ser que receba ajuda.

Pontuação H&Y:_______________________

Teste de velocidade de marcha sem bengala (Uma medida após familiarização)

**Ordem do teste:**	**Tempo (s)**	**Velocidade (m/s)**
Velocidade de marcha auto-selecionada		
Velocidade de marcha máxima		

Episódios de congelamento (parada completa do movimento com posterior retorno) ( )Não ( ) Sim Número de episódios: ______________
Tempo de congelamento: ________

Número de tropeços (perda de equilíbrio com recuperação não assistida): _______

Teste de velocidade de marcha <u>com bengala</u> (Uma medida após familiarização)

**Ordem do teste:**	**Tempo (s)**	**Velocidade (m/s)**
Velocidade de marcha auto-selecionada		
Velocidade de marcha máxima		

Episódios de congelamento (parada completa do movimento com posterior retorno) ( )Não ( ) Sim Número de episódios: ______________
Tempo de congelamento: ________

Número de tropeços (perda de equilíbrio com recuperação não assistida): _______

Teste Timed up and Go- TUG <u>sem bengala</u> (uma repetição após familiarização)
Ordem do teste:______
Tempo:_________________________________ seg
Número de episódios de congelamento: _____________
Tempo de congelamento:
Número de tropeços: ____________________

Teste Timed up and Go- TUG <u>com bengala</u> (uma repetição após familiarização)
Ordem do teste:_____________
Tempo:_________________________________ seg
Número de episódios de congelamento: _____________
Tempo de congelamento:
Número de tropeços: ____________________

AUTOPERCEPÇÃO DE SAÚDE

Em geral, você diria que sua saúde é:

Excelente 1
Muito boa... 2
Boa.. 3
Ruim... 4
Muito Ruim.. 5

Comparada há um ano atrás, como você classificaria sua saúde em geral, agora?

Muito melhor agora do que há um ano atrás............. 1
Um pouco melhor agora do que há um ano atrás..... 2
Quase a mesma coisa do que há um ano atrás........ 3
Um pouco pior agora do que há um ano atrás.......... 4
Muito pior agora do que há um ano atrás...................5

“Em comparação com outras pessoas da sua idade, você diria que sua saúde é:”

1.( ) Melhor 2.( ) Igual 3. ( ) Pior

ESCALA DE CONGELAMENTO DA MARCHA (FOGQ)

1. **Durante o seu pior estado você anda:**

0 Normalmente
1 Quase normalmente – um pouco lento
2 Devagar mas totalmente independente
3 Precisa de ajuda ou de um aparelho para andar
4 Incapaz de andar

2. **Suas dificuldades para andar estão afetando suas atividades de vida diária ou a sua independência?**

0 Nem um pouco
1 Um pouco
2 Moderadamente
3 Severamente
4 Incapaz de andar

3. **Você sente que seus pés estão grudados no chão enquanto você anda, vira ou quandotenta começar a andar (congelamento)?**

0 Nunca
1 Muito raramente – uma vez por mês
2 Raramente – uma vez por semana
3 Frequentemente – uma vez por dia
4 Sempre – toda vez que anda

4. **Quanto tempo dura seu maior episodio de congelamento?**

0 Nunca aconteceu
1 1 a 2 segundos
2 3 a 10 segundos
3 11 a 30 segundos
4 Incapaz de andar por mais de 30 segundos

5. **Quanto tempo dura seu típico episodio de hesitação para começar a andar(congelamento para dar o primeiro passo)?**

0 Nada
1 Leva mais que 1 segundo para começar a andar
2 Leva mais que 3 segundos para começar a andar
3 Leva mais que 10 segundos para começar a andar
4 Leva mais que 30 segundos para começar a andar

6. **Quanto tempo dura sua típica hesitação para virar (congelamento enquanto vira)?**

0 Nada
1 Em torno de 1 a 2 segundos
2 Em torno de 3 a 10 segundos
3 Em torno de 11 a 30 segundos
4 Incapaz de realizar a virada por mais de 30 segundos.

Modified Gait Efficacy Scale (mGES-Brasil)

1- Quanta confiança você tem de que seria capaz de caminhar com segurança sobre uma superfície plana, como um piso de madeira?

1	2	3	4	5	6	7	8	9	10

2- Quanta confiança você tem de que seria capaz de caminhar com segurança na grama?

1	2	3	4	5	6	7	8	9	10

3- Quanta confiança você tem de que seria capaz de passar com segurança sobre um obstáculo no seu caminho?

1	2	3	4	5	6	7	8	9	10

4- Quanta confiança você tem de que seria capaz de descer de um meio fio com segurança?

1	2	3	4	5	6	7	8	9	10

5- Quanta confiança você tem de que seria capaz de subir em um meio fio com segurança?

1	2	3	4	5	6	7	8	9	10

6- Quanta confiança você tem de que seria capaz de subir escadas com segurança, se você estiver segurando em um corrimão?

1	2	3	4	5	6	7	8	9	10

7 Quanta confiança você tem de que seria capaz de descer escadas com segurança, se você estiver segurando em um corrimão?

1	2	3	4	5	6	7	8	9	10

8- Quanta confiança você tem de que seria capaz de subir escadas com segurança, se você NÃO estiver segurando em um corrimão?

1	2	3	4	5	6	7	8	9	10

9- Quanta confiança você tem de que seria capaz de descer escadas com segurança, se você NÃO estiver segurando em um corrimão?

1	2	3	4	5	6	7	8	9	10

10- Quanta confiança você tem de que seria capaz de caminhar com segurança por uma longa distância, como 800 metros (oito quarteirões,aproximadamente)?

1	2	3	4	5	6	7	8	9	10

Nenhuma confiança Total confiança

ESCORE TOTAL

TESTE DE CAMINHADA DE SEIS MINUTOS SEM BENGALA (TC6):
ORDEM DO TESTE: _______

**Antes**	**Após**
PA inicial (mmHg): _________________	PA final (mmHg): __________________
FC inicial (bpm): ____________	FC final (bpm): ____________
FR inicial (rpm): __________________	FR final (rpm): __________________
SpO_2 inicial (%): __________________	SpO_2 final (%): __________________
Fadiga MMII (Borg): __________________	Fadiga MMII (Borg): _______________
Dispneia (Borg): __________________	Dispneia (Borg): __________________

**Teste**	**FC (bpm)**	**SpO2 (%)**	**Dispneia (Borg)**	**Fadiga MMII (borg)**	**Distância (m)**
**1º minuto**					Não se aplica
**2º minuto**					
**3º minuto**					Não se aplica
**4º minuto**					Não se aplica
**5º minuto**					Não se aplica
**6º minuto**					

Parou/pausa antes de seis minutos? ( ) Não ( ) Sim
Motivo:________________Por quanto tempo?
____
Outros sintomas: ________
Distância em **seis** minutos (m):_________
Distância em **dois** minutos (m): _________

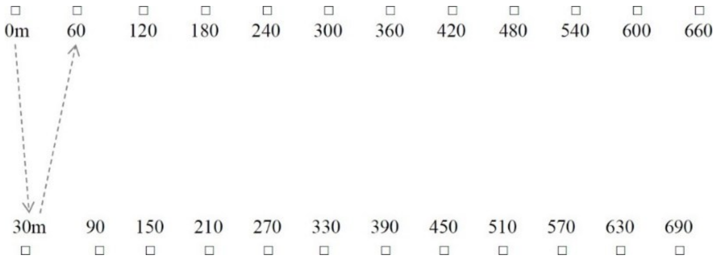

TESTE DE CAMINHADA DE SEIS MINUTOS COM BENGALA (TC6):
ORDEM DO TESTE: _______

**Antes**	**Após**
PA inicial (mmHg): _________________	PA final (mmHg): __________________
FC inicial (bpm): ____________	FC final (bpm): ____________
FR inicial (rpm): __________________	FR final (rpm): __________________
SpO_2 inicial (%): __________________	SpO_2 final (%): __________________
Fadiga MMII (Borg): __________________	Fadiga MMII (Borg): _______________
Dispneia (Borg): __________________	Dispneia (Borg): __________________

**Teste**	**FC (bpm)**	**SpO2 (%)**	**Dispneia (Borg)**	**Fadiga MMII (borg)**	**Distância (m)**
**1º minuto**					Não se aplica
**2º minuto**					
**3º minuto**					Não se aplica
**4º minuto**					Não se aplica
**5º minuto**					Não se aplica
**6º minuto**					

Parou/pausa antes de seis minutos? ( ) Não ( ) Sim
Motivo:________________Por quanto tempo?
____
Outros sintomas: ________
Distância em **seis** minutos (m):_________
Distância em **dois** minutos (m): _________

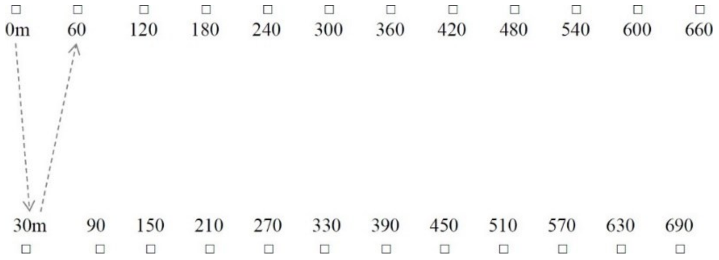

PDQ-39 - QUESTIONÁRIO SOBRE A DOENÇA DE PARKINSON

POR SER PORTADOR DA DOENÇA DE PARKINSON, com que freqüência osenhor/asentiu os seguintes, durante o último mês?

Assinale um quadradinho para cada questão

	Nunca	De vez em quando	Às vezes	Frequen-temente	Sempre/ é impossível para mim
1. Teve dificuldades paraparticipar de atividadesrecreativas que gosta de fazer?					
2. Teve dificuldades para cuidar de sua casa (por ex., fazerpequenos consertos, trabalho de casa, cozinhar)?					
3. Teve dificuldades para carregar sacolas de compras?					
4. Teve problemas para andar um quilômetro (10quarteirões)?					
5. Teve problemas para andar 100 metros (1 quarteirão)?					
6. Teve problemas para se movimentar pela casa com afacilidade que gostaria?					
7. Teve dificuldades para se movimentar em locais públicos?					
8. Necessitou de alguém para acompanhá-lo ao sair?					
9. Sentiu-se assustado ou preocupado com medo de cair empúblico?					
10. Ficou sem sair de casa mais do que gostaria?					
11. Teve dificuldades para se lavar?					
12. Teve dificuldades para se vestir?					
13. Teve dificuldades para abotoar roupas ou amarrarsapatos?					
14. Teve problemas para escrever de maneira legível?					
15. Teve dificuldades para cortar a comida?					
16. Teve dificuldades para segurar uma bebida sem derramar?					
17. Sentiu-se deprimido/a?					
18. Sentiu-se isolado/a e só?					
19. Sentiu que poderia começar a chorar facilmente?					
20. Sentiu-se com raiva ou amargurado/a?					
21. Sentiu-se ansioso/a?					
22. Sentiu-se preocupado/a com seu futuro?					
23. Houve necessidade de esconder sua doença de Parkinsondas outras pessoas?					
24. Evitou situações em que tivesse que comer ou beber empúblico?					
25. Sentiu-se envergonhado/a em público por ter a doença deParkinson?					

26. Sentiu-se preocupado/a com as reações de outraspessoas?					
27. Teve problemas de relacionamento com as pessoas maispróximas?					
Faltou apoio que precisava por parte do seu/sua esposo ou companheiro (a)? Se não tem esposo (a) ou companheiro (a), assinale aqui ( )					
29. Faltou apoio que precisava por parte de sua família ouamigos?					
30. Adormeceu inesperadamente durante o dia?					
31. Teve problemas de concentração, por ex. ao ler ou aoassistir à televisão?					
32. Sentiu que sua memória estava ruim?					
33. Teve sonhos perturbadores ou alucinações?					
34. Teve dificuldades para falar?					
35. Sentiu-se incapaz de comunicar-se com clareza com aspessoas?					
36. Sentiu-se ignorado por outras pessoas?					
37. Teve cãibras musculares dolorosas ou espasmos?					
38. Teve dores nas articulações ou em outras partes docorpo?					
39. Sentiu-se desconfortavelmente quente ou frio?					

Pontuação para cada dimensão
Cada dimensão é calculada em uma escala de 0 a 100:
0 = sem problema; 100 = máximo nível de problema
Fórmula para pontuar cada dimensão
Soma dos escores de cada questão da dimensão x 100
4 (max. escore por questão) x nº questões na dimensão
Dimensões
Mobilidade: 10 questões – 1 a 10
(escores das questões 1+2+3+4+5+6+7+8+9+10) / (4 x 10) x 100
Atividade de vida diária (AVDs):
(escores das questões 11+12+13+14+15+16) / (4 x 6) x 100
Bem estar emocional: 6 questões – 17 a 22
(escores das questões 17+18+19+20+21+22) / (4 x 6) x 100
Estigma: 4 questões – 23 a 26
(escores das questões 23+24+25+26) / (4 x 4) x 100
Suporte Social: 3 questões – 27 a 29
(escores das questões 27+28+29) / (4 x 3) x 100
nota: se o participante indicar que não tem esposo(a)/companheiro(a) a questão 28 pode ser calculado desta forma:
Suporte social: (escores das questões 27+29) / (4 x 2) x 100
Cognição: 4 questões – 30 a 33
(escores das questões 30+31+32+33) / (4 x 4) x 100
Comunicação: 3 questões – 34 a 36
(escores das questões 34+35+36) / (4 x 3) x 100
Desconforto corporal: 3 questões – 37 a 39
(escores das questões 37+38+39) / (4 x 3) x 100

AVALIAÇÃO DA SATISFAÇÃO DO USUÁRIO COM A TECNOLOGIA ASSISTIVA DE QUEBEC B-QUEST (2.0)

Para cada um dos 12 itens, avalie sua satisfação com o re- curso de tecnologia assistiva e os serviços relacionados que experimentou, usando a seguinte escala de 1 a 5:

1	2	3	4	5
Insatisfeito	Pouco satisfeito	Mais ou menos satisfeito	Bastante satisfeito	Totalmente satisfeito

Em caso de algum item com o qual você não tenha ficado “totalmente satisfeito”, comente na seção **comentários**.

1	2	3	4	5
Insatisfeito	Pouco satisfeito	Mais ou menos satisfeito	Bastante satisfeito	Totalmente satisfeito

RECURSO DE TECNOLOGIA ASSISTIVA
Qual é o seu grau de satisfação com:

1. as dimensões (tamanho, altura, comprimento, largura) do seu recurso de tecnologia assistiva?
Comentários: 1 2 3 4 5
2. o peso do seu recurso de tecnologia assistiva?
Comentários: 1 2 3 4 5
3. a facilidade de ajustar (fixar, afivelar) as partes do seu recurso de tecnologia assistiva?
Comentários: 1 2 3 4 5
4. a estabilidade e a segurança do seu recurso de tecnologia assistiva?
Comentários: 1 2 3 4 5
5. a durabilidade (força e resistência ao desgaste) do seu recurso de tecnologia assistiva?
Comentários: 1 2 3 4 5
6. a facilidade de uso do seu recurso de tecnologia assistiva?
Comentários: 1 2 3 4 5
7. o conforto do seu recurso de tecnologia assistiva?
Comentários: 1 2 3 4 5

1	2	3	4	5
Insatisfeito	Pouco satisfeito	Mais ou menos satisfeito	Bastante satisfeito	Totalmente satisfeito

RECURSO DE TECNOLOGIA ASSISTIVA
Qual é o seu grau de satisfação com: (continuação)

a eficácia do seu recurso de tecnologia assistiva (o quanto seu recurso atende às suas necessidades)?
Comentários: 1 2 3 4 5

SERVIÇOS
Qual é o seu grau de satisfação com:

9. o processo de entrega (procedimentos, tempo de espera) pelo qual você obteve o seu recurso de tecnologia assistiva?
Comentários: 1 2 3 4 5
10. os reparos e a assistência técnica (manutenção) prestados para o seu recurso de tecnologia assistiva?
Comentários: 1 2 3 4 5
11. a qualidade dos serviços profissionais (informações, atenção) que você recebeu pelo uso do seu recurso de tecnologia assistiva?
Comentários: 1 2 3 4 5
12. os serviços de acompanhamento (serviços de suporte contínuos) recebidos para o seu recurso de tecnologia assistiva?
Comentários: 1 2 3 4 5

A seguir, consta uma lista com os mesmos 12 itens de sa- tisfação. ESCOLHA OS 3 ITENS que você considera os mais importantes. Assinale um X nas 3 opções de sua escolha.

1) Dimensões □
2) Peso □
3) Ajustes □
4) Segurança □
5) Durabilidade □
6) Facilidade de uso □
7) Conforto □
8) Eficácia □
9) Entrega □
10) Reparos/assistência técnica □
11) Serviços profissionais □
12) Serviços de acompanhamento □

· Número de respostas inválidas ___

· Pontuação subtotal de **Recurso __**

Nos itens de 1 a 8, acrescente a pontuação das respostas válidas e divida essa soma pelo número de itens válidos nesta escala.

· Pontuação subtotal de **Serviços**

Nos itens de 9 a 12, acrescente a pontuação das respostas válidas e divida essa soma pelo número de itens válidos nesta escala.

· Total QUEST ________________

Nos itens de 1 a 12, acrescente a pontuação das respostas válidas e divida esta soma pelo número de itens válidos.

· Os três itens mais importantes de satisfação:

Diário de uso da bengala durante o treinamento

Este diário deverá ser preenchido **todos os dias**. Nele você deverá assinalar com um “x” todos os ambientes em que você utilizou a bengala para se deslocar (caminhar).

Marque se você utilizou a bengala para de deslocar nessas situações

DIAS	01	02	03	04	05	06	07	08	09	10	11	12	13	14	15	16	17	18	19	20
Dentro da sua casa (quartos, sala, etc)																				
No jardim, quintal, varanda da sua casa																				
Para caminhar na rua próximo a sua casa																				
Para se deslocar a locais distantes da sua casa																				
Para subir ou descer escadas e/ou rampas																				

Marque se você caiu em alguma dessas situações

DIAS	01	02	03	04	05	06	07	08	09	10	11	12	13	14	15	16	17	18	19	20
Dentro da sua casa (quartos, sala, etc)																				
No jardim, quintal, varanda da sua casa																				
Para caminhar na rua próximo a sua casa																				
Para se deslocar a locais distantes da sua casa																				
Para subir ou descer escadas e/ou rampas																				
Dentro da sua casa (quartos, sala, etc)																				
**Dias de treinamento**																				

Início do treinamento:____________ **Fim do treinamento:**______________

Diário de uso da bengala

Este diário deverá ser preenchido **todos os dias**. Nele você deverá assinalar com um "x" todos os ambientes em que você utilizou a bengala para se deslocar.

Marque se você utilizou a bengala para de deslocar ne situações

DIAS	01	02	03	04	05	06	07	08	09	10	11	12	13	14	15	16	17	18	19	20	21	22	23	24	25	26	27	28	29	30
Dentro da sua casa (quartos, sala, etc)																														
No jardim, quintal, varanda da sua casa																														
Para caminhar na rua, próximo a sua casa																														
Para se deslocar a locais distantes da sua casa																														
Para subir ou descer escadas e/ou rampas																														

Marque se você caiu em alguma dessas situações

DIAS	01	02	03	04	05	06	07	08	09	10	11	12	13	14	15	16	17	18	19	20	21	22	23	24	25	26	27	28	29	30
Dentro da sua casa (quartos, sala, etc)																														
No jardim, quintal, varanda da sua casa																														
Para caminhar na rua, próximo a sua casa																														
Para se deslocar a locais distantes da sua casa																														
Para subir ou descer escadas e/ou rampas																														
